# Integrating Multi-Omics Atlas to Uncover Genetic and Epigenetic Mechanisms and Reveal Cell State Evolution Across Ecotypes in Male Urological Cancers

**DOI:** 10.3390/ijms27062712

**Published:** 2026-03-16

**Authors:** Jing Bai, He Yu, Congxue Hu, Yining Ma, Mingjie Dong, Liyuan Li, Kaiyue Yang, Zhenzhen Wang, Yunpeng Zhang, Xia Li, Yan Cao

**Affiliations:** 1 College of Bioinformatics Science and Technology, Department of Urology, Harbin Medical University, Harbin 150081, China; baijing@hrbmu.edu.cn (J.B.); 2020020465@hrbmu.edu.cn (H.Y.); hucx1996@hrbmu.edu.cn (C.H.); 2023020587@hrbmu.edu.cn (Y.M.); 2023020613@hrbmu.edu.cn (M.D.); 2022020560@hrbmu.edu.cn (L.L.); yangkaiyue0904@hrbmu.edu.cn (K.Y.); zhangyp@hrbmu.edu.cn (Y.Z.); 2 School of Intelligent Medicine and Technology (Big Data Research Center), Hainan Medical University, Haikou 571199, China; wangzhenzhen@hainmc.edu.cn

**Keywords:** male urological cancers, epigenetics, cancer ecotype, spatial transcriptomics, single-cell RNA sequencing

## Abstract

Male urological cancers, including clear cell renal cell carcinoma (ccRCC), bladder cancer (BC), and prostate cancer (PCa), are characterized by extensive heterogeneity and complex ecosystems, yet the underlying mechanisms remain incompletely understood. In this study, scRNA-seq, scATAC-seq and spatial transcriptomics data are integrated to systematically characterize the features of the tumor microenvironment (TME). We identify tumor cell subclones and elucidate the impact of chromosomal abnormalities on their characteristic functions. We further identify transcription factor regulatory networks by analyzing tumor cell differentiation trajectories. Importantly, we integrate DNA methylation and SNP information to deeply dissect the tumor cell differentiation process, revealing the multilayer regulatory mechanisms of tumor-related genes. Additionally, we reveal the evolution of cellular states across ecotypes to provide a more comprehensive characterization of TME. Finally, we screened potential therapeutic agents targeting the molecular mechanisms underlying tumor cell differentiation (Amivantamab in ccRCC, Levothyroxine in BC, Ouabain in PCa) and signature genes of ecotype. In conclusion, our work establishes a comprehensive framework for tumor assessment and informs the development of precision therapeutic strategies.

## 1. Introduction

Male urological cancers primarily encompass cancers of the bladder, kidneys, and prostate. Clear cell renal cell carcinoma (ccRCC), bladder cancer (BC) and prostate cancer (PCa) are heterogeneous diseases, with both their incidence and mortality rates showing concerning trends [1]. PCa is one of the most common cancers in men [2]. Males exhibit a high incidence of renal cancer and BC [3,4,5]. Therefore, a systematic analysis of both the specific and shared characteristics of male urological cancers is urgently needed. A comprehensive understanding of their genetic and epigenetic mechanisms, as well as the tumor microenvironment (TME), may facilitate the identification of novel therapeutic targets.

With the advent of single-cell RNA sequencing (scRNA-seq) and single-cell ATAC sequencing (scATAC-seq), research at single-cell resolution has advanced rapidly [6,7], providing powerful technical tools for revealing tumor cell characteristics and the TME [8,9,10]. Current evidence suggests that accumulated genetic alterations and shifts in expression patterns drive cancer progression, leading to a cascade of histopathological changes [11]. Both copy number variations (CNVs) and single-nucleotide polymorphisms (SNPs) may serve as causal events that initiate and promote cancer development. Moreover, a mounting body of evidence in recent years has indicated that epigenetics plays a pivotal role in various aspects of disease progression [12]. Therefore, integrating scRNA-seq and scATAC-seq data enables a comprehensive dissection of genetic alterations and epigenetic dysregulation across distinct cellular subsets during malignant progression.

In addition, tumor tissues possess a complex ecosystem primarily composed of tumor cells, immune cells, and stromal cells, embedded within an altered extracellular matrix [13]. Within this microenvironment, diverse cell types and states organize into distinct ecotypes [14,15]. The interactions within ecotypes play a crucial role in tumor progression [16]. Furthermore, spatial transcriptome sequencing enables the visualization and quantitative analysis of the transcriptome at each spot within tissue sections [17]. In this study, we incorporate spatial transcriptome into our research framework to investigate the molecular and spatial features of tumor ecotypes.

In summary, we integrated scRNA-seq, scATAC-seq and spatial transcriptomics data to systematically characterize the TME of male urological cancers. We uncovered the genetic and epigenetic mechanisms underlying tumor cell differentiation. Moreover, we investigated the functional characteristics of distinct ecotypes and the evolution of cellular states across ecotypes. Finally, we identified drugs targeting the molecular mechanisms underlying tumor cell differentiation and signature genes of ecotypes. Our work establishes a comprehensive framework for evaluating male urological cancers and provides valuable insights for the development of precise therapeutic strategies.

## 2. Results

### 2.1. Multi-Omics Atlas of Male Urological Cancers

**Figure 1:**
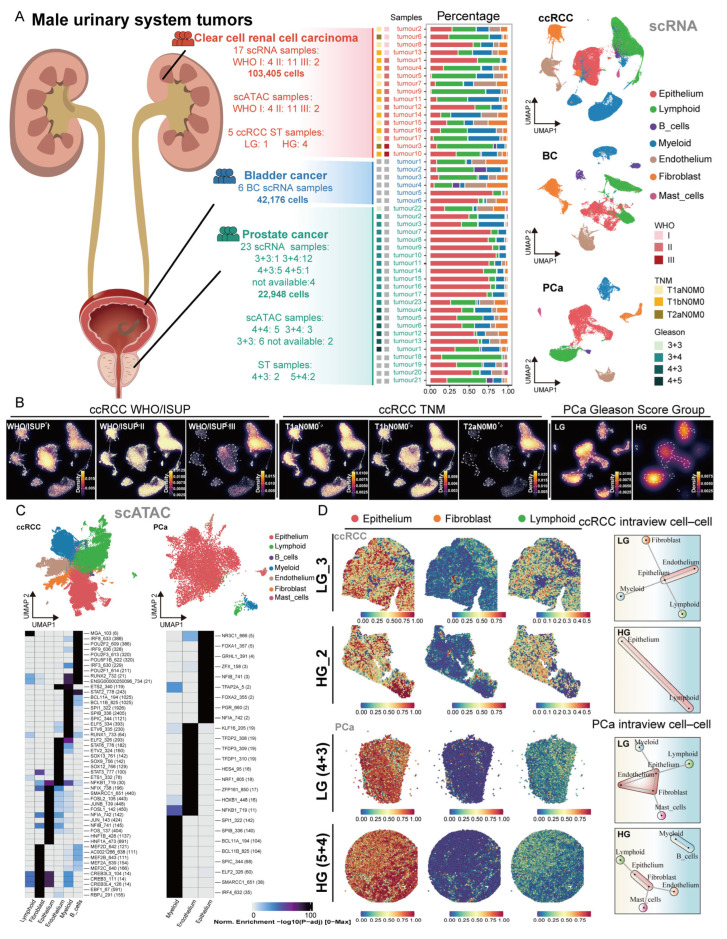
Single-cell atlas of male urological cancers. (**A**) (**Left**): Schematic illustration of male urological cancers (ccRCC, BC, and PCa). (**Center**): Bar plot showing the proportion of cell types in each sample. (**Right**): The scRNA-seq atlas of male urological cancers. (**B**) Cell density plots derived from scRNA-seq data, stratified according to WHO/ISUP grades (I, II, III) and TNM stages for ccRCC, and Gleason score groups for PCa. (**C**) The top panel displays the scATAC-seq landscapes of ccRCC and PCa, while the bottom panel shows motifs that are differentially enriched across distinct cell types (p-adj < 0.05). (**D**) The left panel shows the abundance of each cell type within spatial transcriptomics spots, while the right panel shows the dependencies between different cell types inferred from different pathological groups.

To investigate the heterogeneity of male urological cancers, we performed scRNA analysis on ccRCC, BC, and PCa. In this study, we collected scRNA-seq data comprising 103,405 cells from 17 ccRCC samples, 42,176 cells from 6 BC samples, and 22,948 cells from 23 PCa samples. Additionally, we acquired scATAC-seq data from 9142 cells across 15 PCa samples and 116,733 cells from 17 ccRCC samples, along with spatial transcriptomic profiles from 4 PCa and 5 ccRCC specimens (Table S1). PCa samples with Gleason scores of 4+4, 4+5 and 5+4 were classified into the high Gleason score group (HG), whereas samples with Gleason scores of 3+3, 3+4 and 4+3 were classified into the low Gleason score group (LG). Subsequently, we constructed a comprehensive multi-omics atlas of male urological cancers, which served as the foundation for subsequent analyses (Figure S1A). Utilizing canonical markers of cell types based on scRNA data, we identified a total of 7 distinct cell types, including epithelial cells, stromal cells (endothelial cells and fibroblasts), and immune cells (lymphoid cells, myeloid cells, mast cells and B cells) (Figure 1A, Figures S1B and S2A). We observed the cell density through the WHO/ISUP grades and TNM stages in ccRCC and the Gleason score groups in PCa (Figure 1B). We observed the presence of immune cells, including lymphoid cells and myeloid cells, across WHO/ISUP grades (I, II and III) of ccRCC. Similarly, lymphoid cells and myeloid cells were detected in PCa samples across different Gleason score groups. To further evaluate immune infiltration, we applied xCell to bulk RNA-seq data and calculated immune scores for samples from each pathological group (Figure S2B). The results showed that ccRCC tumor tissues exhibited higher immune scores than normal tissues. PCa tumor tissues also displayed elevated immune scores compared with normal tissues, suggesting that both ccRCC and PCa harbor immune infiltration.

Label transfer analysis in scATAC-seq data revealed distinct cell types, including epithelial cells, endothelial cells, fibroblasts, lymphoid cells, myeloid cells and mast cells (Figure 1C). ArchR was used to identify cell-type marker peaks, thereby revealing epigenetic differences among cell types. We then further explored the motifs enriched in these cell-type marker peaks (Figure 1C and Figure S2D). We observed a significant enrichment of the FOSL1 motif in the epithelial cells of ccRCC. In addition, we observed a significant enrichment of the FOXA1 motif in epithelial cells of PCa. Epithelial cells in ccRCC and PCa were enriched for the motifs of NFIB and NFIA, whereas endothelial cells were specifically enriched for NFKB1 motif (Figure 1C). Additionally, we inferred TF activity from scRNA-seq data using decoupleR (Figure S3A,B). We identified the transcription factors (TFs) SPIC and SPIB with high activity in myeloid cells across all three cancers. These findings indicate that the same cell types in male urological cancers show enrichment of some similar TFs, suggesting some shared regulatory mechanisms.

The differences in cellular composition in male urological cancers, as well as the role of epithelial cell TFs in cancer, prompted us to further investigate the spatial characteristics of cell types. By inferring the abundance of cell types in spots using seurat, we demonstrated the predominant enrichment of epithelial cells across most spatial spots (Figure 1D and Figure S3C–E). Using the MISTy framework, we identified distinct spatial co-localization patterns across cancer types. Specifically, ccRCC exhibited co-occurrence of epithelial cells with lymphoid cells, while PCa demonstrated proximal epithelial-fibroblast interactions with distal lymphoid cells positioning (Figure 1D).

These results provide preliminary insights into the cellular composition of male urinary cancers. Same cell types show enrichment of some similar TFs. The spatial distribution highlights the dominant role of epithelial cells and their interdependence with other cells. These enhance the micro-level understanding of the TME in male urological cancers.

### 2.2. Identification and Functional Characterization of Tumor Cell Subclones in Male Urological Cancers

**Figure 2:**
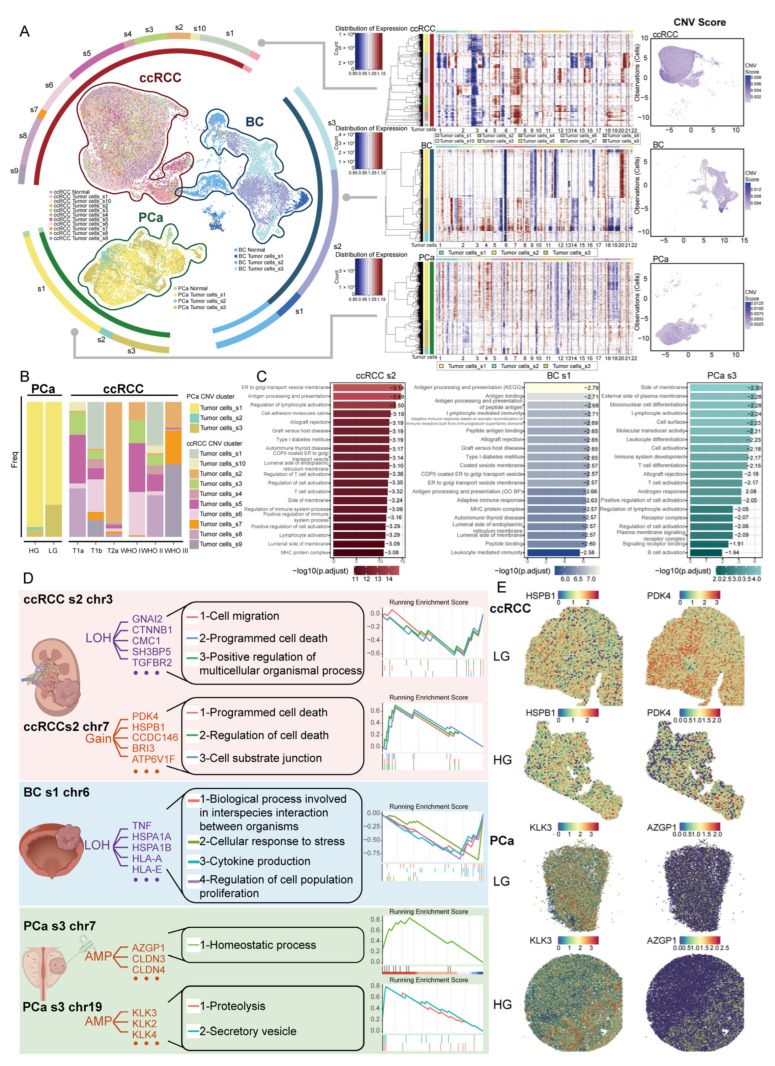
CNV landscape of epithelial cells in male urological cancers. (**A**) Left: Clustering of normal epithelial cells and tumor subclones in male urological cancers, with surrounding bar plots showing the proportions of each cell population. Center: CNV landscape across tumor subclones. Red and blue colors represent high and low CNV levels, respectively. Right: CNV scores for individual cells. (**B**) Bar plots showing the proportions of tumor subclones across WHO/ISUP grades and TNM stages in ccRCC, and Gleason score groups in PCa. (**C**) Bar plot showing the results of functional enrichment analysis for CNV genes in tumor subclones ccRCC s2, BC s1, and PCa s3. (**D**) GSEA enrichment analysis results of amplified and deleted genes on chromosomes in tumor subclones from different cancers. (**E**) Expression of the CNV gene in the spatial transcriptome.

Chromosomal instability serves as a driving force in cancer development [18], and it can be characterized by CNVs. Using scRNA-seq data, we first distinguished tumor cells from their normal counterparts through CNV profiling and subsequently reconstructed tumor-specific CNV landscapes (Figure S4A). There are significant differences in amplification and deletion in different genomic regions among cancer types (Figure 2A). We further examined the functional enrichment of genes associated with CNVs in cancer (Figure S4B). The results show that in male urological cancers, CNVs affect the interferon response and inflammatory functions. Interestingly, CNV-associated genes in PCa were primarily enriched in functions related to androgen response. This suggests that CNVs play a role in cancer heterogeneity and may influence therapeutic approaches.

Tumor cells were classified into distinct subclones based on CNV patterns, with each subclone exhibiting different proportions across various pathological subgroups of the cancer. By identifying DEGs between tumor subclones and normal epithelial cells, we further filtered the inferred CNV-associated genes. These included PBRM1, SETD2, and KDM5C in BC; PPARG, EGFR, CCND1, and CDKN2A in ccRCC; and AR and CDKN1B in PCa (Figure S4C and Table S2).

We found that certain tumor subclones could be identified across multiple patients, such as subclones s2 and s3 in ccRCC, subclone s1 in BC, and subclones s1 and s3 in PCa. In contrast, some tumor subclones exhibited patient-specific patterns, including ccRCC subclone s1 and BC subclone s2 (Figure S4D). BC subclone s1 harbors CNV-associated genes that may influence functions related to immune and cell migration (Table S2). In ccRCC subclone s2, which potentially affects functions related to immune and oxidative phosphorylation, is enriched in late-stage tumors (Figure 2B and Table S2). In PCa, CNVs in PCa subclone s3 are associated with the androgen response (Figure 2C and Table S2).

To further analyze the impact of CNVs on subclones, we specifically examined CNV-associated genes across different chromosomes in these subclones and assessed how CNVs on various chromosomes impacted biological functions. We observed notable differences in CNV patterns between males and females from TCGA (Figures S4E and S5A). We further explored the functional impact of CNVs in males and validated the identified CNVs using male TCGA data. In BC subclone s1, genes with copy number loss on chromosome 6 are enriched in cellular response to stress and cytokine production. In PCa subclone s3, genes with copy number gain on chromosome 7 are enriched in the homeostatic process. Furthermore, genes with copy number gain on chromosome 19 are enriched in proteolysis and the secretory vesicle. In ccRCC subclone s2, genes with copy number loss on chromosome 3 are enriched in cell migration and programmed cell death, whereas genes with copy number gain on chromosome 7 are enriched in programmed cell death and cell–substrate junction organization (Figure 2D). We observed that ccRCC subclone s2, the genes with copy number gain, PDK4 and HSPB1, were highly expressed in tumor regions. Similarly, in PCa subclone s3, the amplified genes AZGP1 and KLK3 showed elevated expression specifically in tumor regions (Figure 2E).

We observed that the majority of tumor subclones across all three cancer types exhibited copy number loss on chromosome 6 (Figure S5B). Functional enrichment analysis of genes affected by chromosome 6 deletions in different tumor subclones, as well as genes commonly deleted across all three cancers, indicated that these deletions may impact tumor cell immune responses (Figure S5C,D). In addition, we performed CNV analysis of the X and Y chromosomes in tumor cells (Figure S5E). The analysis revealed that chromosome X exhibited copy number gains across all three cancer types. Functional enrichment of genes with copy number gain on chromosome X showed that these genes are primarily associated with the regulation of protein ubiquitination and regulation of protein modification (Figure S5F).

CNV analysis of tumor cells revealed commonalities and specific features across male urological cancers. Distinct CNV patterns were observed among different tumor subclones. Additionally, loss on chromosome 6 impairs immune responses, and gain on chromosome X impairs protein ubiquitination. These results enhance our understanding of how CNVs within subclones contribute to the occurrence and progression of male urological cancers.

### 2.3. Deciphering the Transcriptional Regulatory Programs Driving Tumor Cell Differentiation

Tumor cells typically originate from epithelial cells, and cancers are closely associated with changes in gene transcription in epithelial cells [19]. We first compared the gene expression profiles between tumor and normal epithelial cells. Tumor cells from distinct cancer types displayed high expression of tumor type-specific marker genes. In PCa, FOLH1 and KLK3 were specifically expressed in tumor cells, while in ccRCC, tumor cells showed specific expression of CA9 (Figure S6A). DEG analysis showed that tumor cells from all three cancers were significantly enriched in functions related to epithelial cell development, response to hypoxia, and negative regulation of the apoptotic signaling pathway, which are closely associated with tumor progression (Figure S6B). Notably, enrichment of response to peptide hormone and renal system development functions was observed, which may represent unique molecular features of male urological cancers.

To further explore transcriptional heterogeneity within epithelial cells, we performed NMF analysis to identify gene modules (Figure S6D and Table S3). We also performed functional enrichment analysis based on the genes within each meta-program (MP). Notably, ccRCC MP1/MP5/MP6, BC MP3, and PCa MP1 all exhibited enrichment of the response to steroid hormone function. In addition, we found that tumor cells in ccRCC were enriched in MP associated with endocrine hormone response (ccRCC MP4) and cell–cell junction organization (ccRCC MP5). In BC, tumor cells were enriched in MP related to the collagen-activated signaling pathway (BC MP3). In PCa, tumor cells showed enrichment in MP associated with transcription (PCa MP5) (Figure S6E).

**Figure 3:**
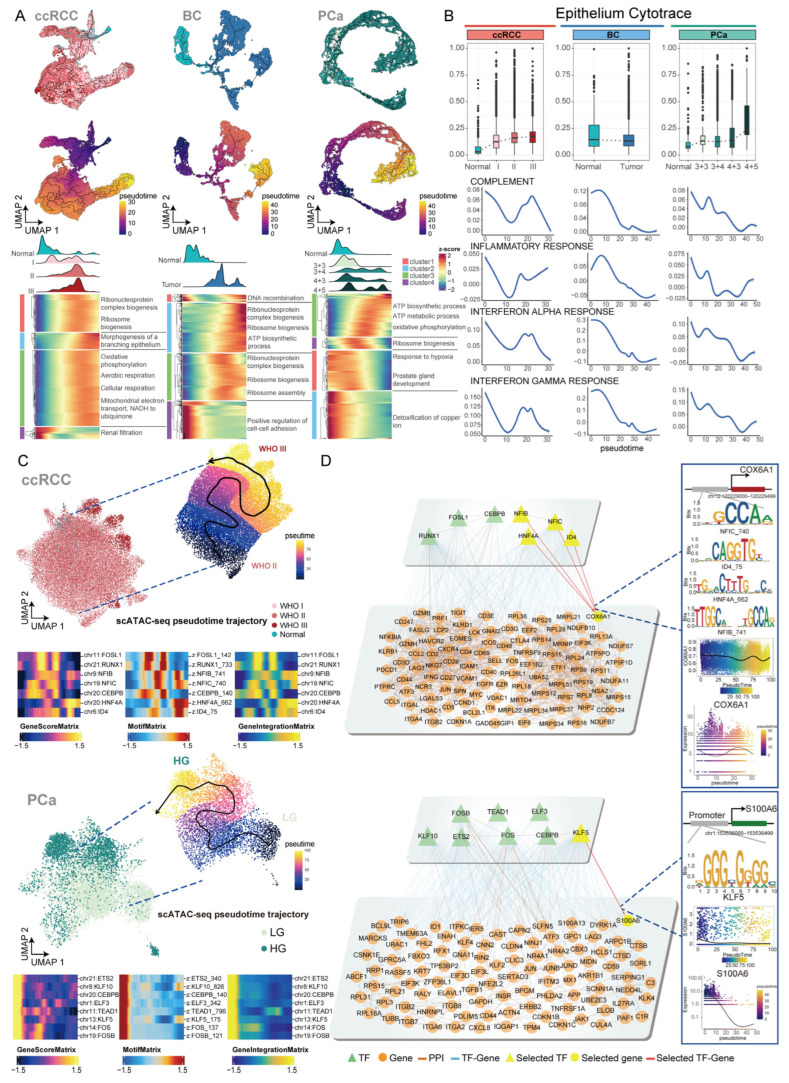
Cellular differentiation from normal epithelial to tumor cell states: (**A**) The top panel displays the Monocle3 differentiation trajectories from normal epithelial to tumor cells in ccRCC, BC, and PCa. The bottom panel shows the differentiation of cells at various pathological grades during the progression, along with the functional annotation of gene clusters involved in the differentiation process. (**B**) The top panel displays cellular stemness across different pathological grades. The bottom panel shows changes in functional gene set scores during the differentiation process. (**C**) The top panel of the upper section shows the pseudotime trajectory of ccRCC tumor cells differentiating from WHO/ISUP grade II to WHO/ISUP grade III based on scATAC-seq data. The dashed lines represent the origins of the pseudotime trajectory, and the arrows indicate the direction of the pseudotime trajectory. The bottom panel of the upper section displays the gene scores of the identified TFs, their corresponding motifs, and the changes in TF expression along the pseudotime trajectory. The top panel of the lower section shows the pseudotime trajectory of ccRCC tumor cells differentiating from WHO/ISUP grade II to WHO/ISUP grade III in scATAC-seq data, while the bottom panel of the lower section illustrates the gene scores of the identified TFs, their corresponding motifs, and the dynamics of TF expression along pseudotime. (**D**) Regulatory networks of TFs involved in driving tumor cell differentiation, alongside protein–protein interaction (PPI) networks among associated genes. The right panel presents gene scores and gene expression during the differentiation process.

We observed that epithelial cell marker genes were enriched for hypoxia-related functions in ccRCC and filament-related functions in PCa and BC (Figure S7 and Table S3). We then constructed pseudotime trajectories to analyze functional changes during tumor cell differentiation (Figure 3A). In ccRCC, epithelial cells differentiated from low WHO/ISUP grade cells to high WHO/ISUP grade cells, while in PCa, epithelial cells differentiated from low Gleason score cells to high Gleason score cells. Through functional enrichment analysis, we found that genes highly expressed in higher pathological grade tumor cells were enriched in functions related to cell growth (Figure S6C). Consistent with these findings, CytoTRACE2 analysis indicated that high-grade tumor cells in both PCa and ccRCC retained intrinsic differentiation potential (Figure 3B). The heatmap showed that during differentiation, tumor cells upregulate functions related to ATP synthesis, which may affect their growth and development (Figure 3A and Figure S8). Additionally, we found that the interferon response score in tumor cells gradually decreased (Figure 3B). In BC and PCa, the complement score decreased (Figure 3B). In ccRCC and PCa, the function of the inflammatory response presents complex changes.

In scATAC data, we also observed that the tumor cell differentiation trajectory in ccRCC shifted from the WHO/ISUP grade II cells to the WHO/ISUP grade III cells, while in PCa, tumor cells differentiated from lower Gleason score cells to the high Gleason score cells (Figure 3C). Subsequently, we further identified key TFs driving the tumor cell differentiation and constructed TF-gene regulatory networks to identify the CNV genes regulated by TF, including CNV genes on chromosomes X and Y (Figure 3D and Figure S9A and Table S3). In ccRCC, we identified 7 TFs (FOSL1, RUNX1, NFIB, NFIC, CEBPB, HNF4A and ID4) that drive tumor cell differentiation. We found that the TFs RUNX1 and HNF4A exhibit higher expression levels in males (Figure S9B). Subsequently, we constructed a regulatory network based on target genes showing higher expression in males (Figure S9C). Under the regulation of NFIC, ID4, HNF4A, and NFIB, the expression of COX6A1 decreases during the differentiation process. In PCa, we identified 8 TFs (ETS2, KLF10, CEBPB, ELF3, TEAD1, KLF5, FOS and FOSB) that drive tumor cell differentiation. The expression level of the S100A6 gene, regulated by the KLF5, gradually decreases during the differentiation process. In addition, we found TFs and their target genes are expressed in close spatial proximity (Figure S9D). These results suggest that the regulatory mechanisms of the TFs we identified may influence cancer progression.

In general, we focused on investigating the molecular mechanisms underlying malignant progression. The results revealed multiple TFs that drive tumor cell differentiation, laying the foundation for a deeper understanding of the molecular mechanisms underlying tumor cell differentiation.

### 2.4. Identifying the Co-Occurrence of Genetic and Epigenetic Alterations During Tumor Cell Differentiation

**Figure 4:**
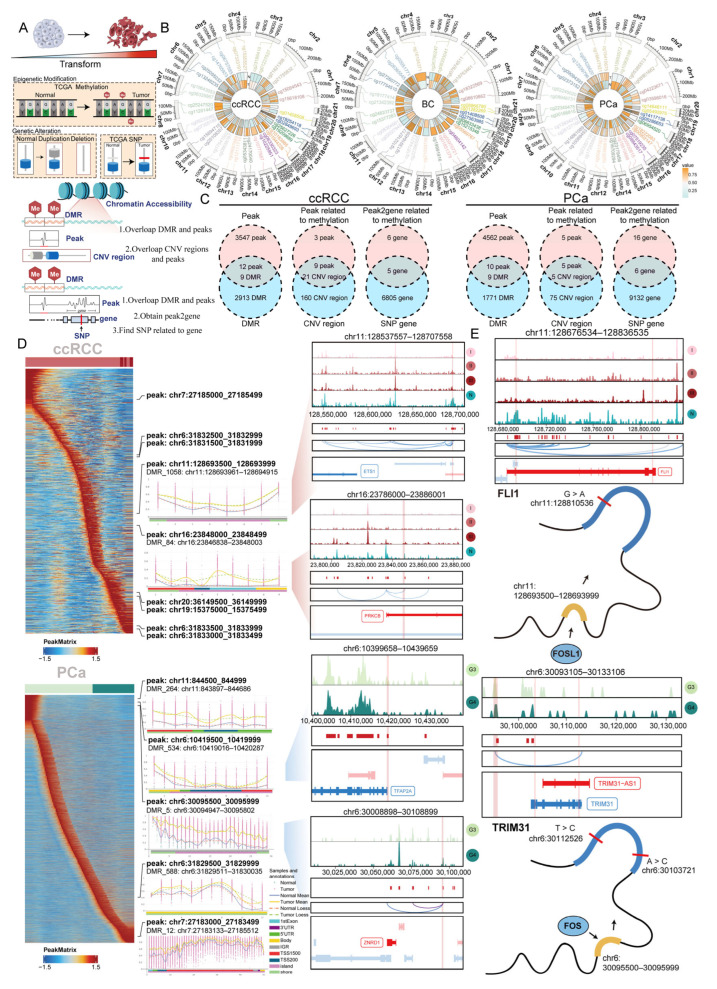
Genetic alterations driving the differentiation process: (**A**) Schematic diagram of dissecting the roles of CNVs, DNA methylation, and SNPs during the differentiation from normal epithelial to tumor cells. In the lower panel, yellow in the DMR indicates methylation, red in the peaks indicates peak overlapped with DMRs, and SNPs with arrows indicate genes associated with the peaks that harbor SNPs. (**B**) Heatmap showing differential DNA methylation between normal and tumor tissues. (**C**) Venn diagrams showing the intersections between CNV regions and methylation regions, as well as between SNP genes and methylation genes. (**D**) The left panel shows peaks with chromatin accessibility changes along pseudotime. The middle panel displays the identified CMRs, with peaks in the upper portion and DMRs overlapping with the peaks in the lower portion. The right panel shows genes associated with peaks. (**E**) Shows genes undergoing SNPs and methylation.

Exploring the mechanisms of epigenetic modifications and genetic alterations during tumor cell differentiation is critically important. Among these, methylation is associated with chromosomal instability and can alter gene expression without changing the underlying DNA sequence [20]. We identified differentially methylated regions (DMRs) between normal and tumor tissues. By integrating these DMRs with CNV regions inferred from scRNA-seq data, we further explored chromatin regions overlapped by both DNA methylation and CNV regions, which we refer to as CNV-associated methylation regions (CMRs). We identified peaks where CNV regions and DNA methylation regions overlap. The peaks identified from overlapping regions were used for the next analysis. Additionally, we performed SNP analysis on genes linked to methylation-related peaks, categorizing genes with SNPs as high-risk genes (Figure 4A).

We collected methylation data from different cancer patients in the TCGA database and identified DMRs between tumor and normal tissues (Figure 4B and Figure S10A–C). We performed functional enrichment analysis on the DMRs identified in the three cancer types. We found that among the differentially methylated genes, the gene set “BENPORATH_ES_WITH_H3K27ME3” showed the highest enrichment (Figure S10D). The results of functional enrichment analysis suggest that methylation impacts tumor cell stemness, which in turn contributes to malignant progression by increasing proliferative and invasive capabilities.

Based on scATAC-seq data, we next overlapped the methylation regions with CNV regions by peaks to identify CMRs. To investigate their transcriptional impact during tumor cell differentiation, we analyzed these regions and identified 9 CMRs in ccRCC and 5 CMRs in PCa (Figure 4C, Figures S11 and S12). Next, we examined the changes in chromatin accessibility of these CMRs along the pseudotime trajectory (Figure 4D). CMRs may regulate gene expression during tumor cell differentiation, thereby influencing cancer progression. For example, in ccRCC, the CMR containing the DNMT1 and IRF2 motifs is linked to the gene ETS1 and may potentially affect its expression, while the CMR containing the NFIC motif is linked to the gene PRKCB and may potentially affect its expression. In PCa, the CMR containing the ELF3 motif is linked to the gene TFAP2A and may potentially affect its expression, while the CMR containing the FOS motif is linked to the gene ZNRD1 and may potentially affect its expression. Further analyses of chromosomes X and Y revealed that in ccRCC, the regulatory peak associated with PRICKLE3 expression is under the regulation of the TF FOSL1 and is concurrently affected by DNA methylation and CNV, indicating a combined transcriptional, epigenetic, and genomic regulation (Table S4).

SNPs are closely associated with DNA methylation [21,22]. Therefore, we integrated SNP data to investigate whether genes affected by CNV and methylation also exhibit SNPs, leading to the identification of high-risk genes (Table S4). For example, in ccRCC, the CMR containing the FOSL1 motif is linked to the gene FLI1 and may potentially affect its expression. The G > A mutation is present in FLI1. In addition, we found that the risk genes THRA and HOXA10, which are subject to multifaceted regulatory mechanisms, show lower expression levels in males compared with females from TCGA (Figure S12B). In PCa, the CMR containing the FOS motif is linked to the gene TRIM31 and may affect its expression. Furthermore, T > C and A > C mutations are present in TRIM31 (Figure 4E).

Our analysis indicates that DNA methylation plays a critical role in tumor malignant progression. Furthermore, we identified genes associated with a high risk of genetic variation by analyzing the impact of SNP data. This deepens our understanding of the molecular mechanisms underlying tumor cell differentiation.

### 2.5. Systematically Characterizing Tumor Molecular and Spatial Ecotypes

**Figure 5:**
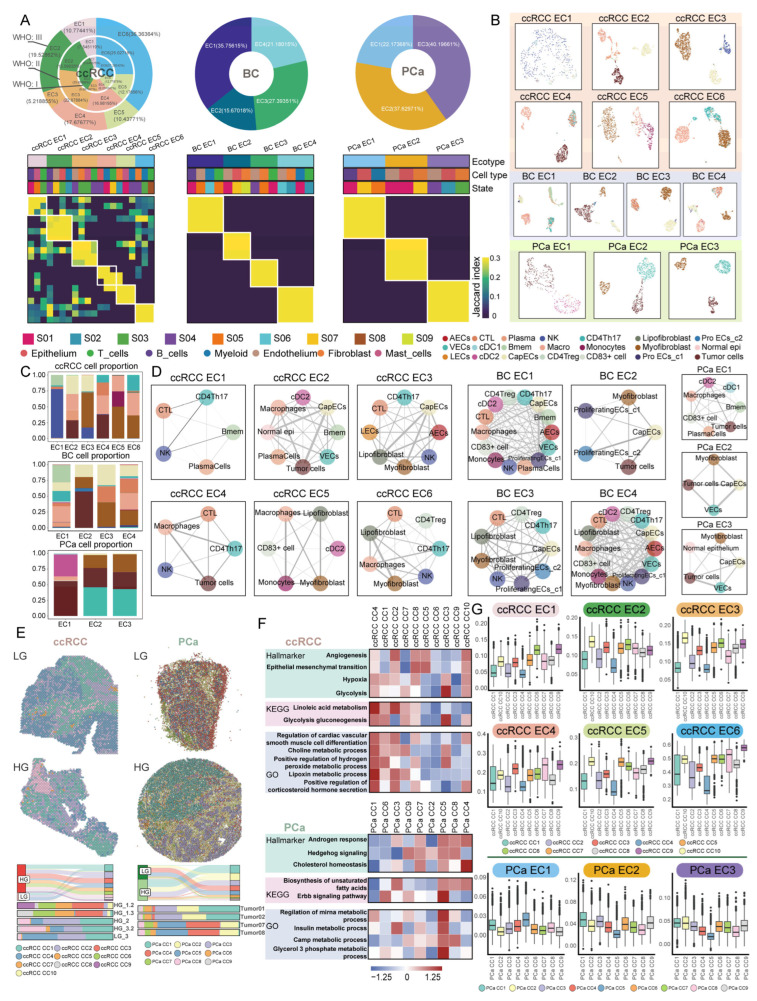
Ecotype atlas of male urological cancers: (**A**) Heatmap displaying the ecotypes identified in ccRCC, BC, and PCa. Pie charts showing the proportions of each ecotype across different pathological grades. (**B**) UMAP plot showing the clustering of cell subpopulations across different ecotypes. (**C**) Bar plots showing the proportions of cell subpopulations across different ecotypes. (**D**) CCI networks among cellular subpopulations within each ecotype (**E**) (**Top**): Spatial localization of spot clusters. (**Bottom**): Proportional representation of spot clusters within various pathological classifications. (**F**) Heatmap illustrating functional enrichment scores in various spatial transcriptomics spot clusters. (**G**) Boxplots showing the scores of different ecotypes across various spot clusters. The colors represent CCs, and each point represents a spot.

Ecotypes (ECs) shape biology and potentially influence the response to therapy in male urological cancers. We first annotated cell subpopulations based on marker genes (Figure S13). Subsequently, we identified the cellular states and inferred the ecotypes (Figure 5A and Figures S14–S16). We identified 6 ecotypes in ccRCC (ccRCC EC1-EC6), 4 ecotypes in BC (BC EC1-EC4) and 3 ecotypes in PCa (PCa EC1-EC3), each characterized by feature genes (Figure 5B). The feature genes of the BC ecotypes were also expressed in an independent BC scRNA-seq dataset (Figure S17A,B).

Functional enrichment analysis was conducted using feature genes of each ecotype (Figure S17C and Table S5). Fibroblasts in ccRCC EC3 and EC5, BC EC2, and PCa EC2 were enriched for genes associated with renal system development, which may represent a characteristic feature of urogenital tumor ecotypes. Additionally, endothelial cells in ccRCC EC2, BC EC3, and PCa EC2 were enriched for the regulation of endothelial cell migration. This indicates that the ecological features of male urogenital cancers share certain similarities (Figure S18A).

In ccRCC, ccRCC EC6 was most abundant in WHO/ISUP grade III, with lymphoid cells primarily involved in T cell differentiation. The proportion of ccRCC EC4 increased progressively with advancing WHO/ISUP grade. Lymphoid cells in this ecotype did not exhibit immune-related signatures. Further analysis revealed that ccRCC EC4 contained tumor cells, the majority of which belonged to ccRCC subclone s8 (Figure 5C and Figure S18B). The ccRCC subclone s8 was primarily associated with functions related to the regulation of T cell activity (Table S2). Through cell–cell interaction analysis, we found that in ccRCC EC4, tumor cells modulated CTL (Cytotoxic T lymphocyte) function through interactions between ligands (NECTIN2, NECTIN3, and PVR) and an immune checkpoint receptor TIGIT (Figure 5D and Table S5). This finding further supports the notion that tumor cells within ccRCC EC4 suppress the cytotoxic activity of CTLs.

In PCa, PCa EC2 and PCa EC3 were the most predominant ecotypes (Figure 5A). Analysis of tumor subclonal composition revealed that tumor cells within these two ecotypes were largely derived from PCa subclone s3, which was primarily associated with cell adhesion-related functions. Moreover, CCI analysis revealed that, in PCa EC2 and PCa EC3, tumor cells interact with myofibroblasts via the EDN1-EDNRA signaling pathway, potentially facilitating tumor cell migration.

In BC EC2, we found that the majority of tumor cells belonged to BC subclone s3. The genes amplified on chromosome 2 in BC subclone s3 are involved in promoting epithelial cell development. Meanwhile, within this ecotype, myofibroblasts interact with tumor cells via the NRG1-ERBB3 signaling pathway. Additionally, fibroblasts in both PCa EC2 and BC EC2 were enriched for functions related to renal system development. We observed that tumor cells in both PCa EC2 and BC EC2 communicate with myofibroblasts through EFNA1–EPHA3 ligand–receptor interactions, which may be associated with tumor progression (Figure 5D).

Observing the characteristics of ecotypes from a spatial perspective can deepen our understanding of them. Therefore, we integrated distinct spatial transcriptomic samples to identify compositional clusters (CCs) (Figure S19). We observed that within the same type of cancer, the proportions of spot clusters varied across different malignant groups (Figure 5E). In ccRCC, ccRCC CC7 was enriched in the low-grade group and primarily associated with metabolic pathways, including choline metabolism and lipoxin metabolism. Both ccRCC CC5 and ccRCC CC3 were enriched in the high-grade group. We found that ccRCC CC3 showed significant enrichment in Glycolysis. And ccRCC CC5 showed significant enrichment in epithelial–mesenchymal transition (EMT) related functions. Furthermore, PCa CC5, which is predominantly enriched in the LG, showed significant enrichment in a range of metabolic pathways, such as cAMP metabolism and the insulin signaling pathway (Figure 5F).

Next, we compared the proportions of different ecotypes across various pathological subgroups. By scoring CCs using feature genes of ecotypes, we found that the proportion of ccRCC CC3 expressing ccRCC EC4 features was higher in the high-grade group, indicating that more tumor cells influence immune cell functions in advanced ccRCC, consistent with observations from scRNA-seq data (Figure 5G). In HG, PCa CC1 and CC3, which are enriched in this group, showed high expression of PCa EC2 and EC3 features, suggesting closer tumor stroma interactions in advanced PCa (Figure 5G).

These results suggest that certain ecotype characteristics are shared across male urological cancers. Tumor cell subclones may influence the functional characteristics of ecotypes. Furthermore, changes in ecotypes are associated with malignant tumor progression, providing a foundation for subsequent mechanistic investigations.

### 2.6. Dissecting Cellular State Evolutionary Trajectories Across Ecotypes

The same cell type can perform different functions across ecotypes, potentially due to differences in their differentiation status. By integrating ecotype information with pseudotime results, we mapped the differentiation processes of various cell types within different ecotypes (Figures S20–S23).

**Figure 6:**
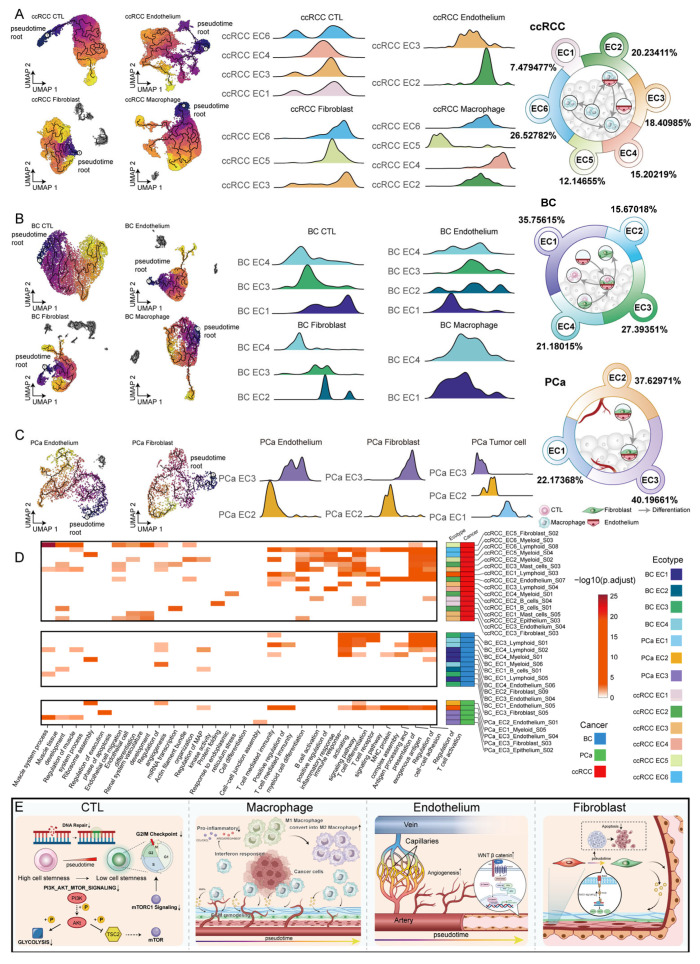
Cellular differentiation trajectory across tumor ecotypes. (**A**–**C**) (**Left**): Monocle3 differentiation trajectories of CTLs, macrophage, endothelial cells and fibroblasts. (**Center**): Distribution of cells from each ecotype during the differentiation process. (**Right**): Schematic diagram of cross-ecotype cellular differentiation trajectories. Symbols represent different cell types, and arrows indicate the direction of differentiation. (**D**) Heatmap illustrating functional enrichment profiles of various cell types within distinct ecotypes. (**E**) Schematic diagram of functional changes in CTLs, macrophages, endothelial cells, and fibroblasts during differentiation.

Through cross-ecotype differentiation trajectories, we found that macrophages first differentiate from ccRCC EC5 into ccRCC EC2 and ccRCC EC6, and finally into ccRCC EC4 (Figure 6A). Myeloid cells in ccRCC EC5 exhibit functional characteristics associated with immune activation, whereas those in ccRCC EC4 are primarily related to lysosome organization (Figure 6D). Through cell–cell interaction analysis, we found that tumor cells in ccRCC EC4 can influence macrophages via the CCL15–CCR1 signaling pathway (Table S5). This indirectly suggests that macrophages exhibit impaired immune function under the influence of tumor cells.

Additionally, we found that endothelial cells in ccRCC EC3 were enriched for functions related to endothelial cell differentiation and exhibited a potential differentiation trajectory toward the endothelial cell state observed in ccRCC EC2. Endothelial cells in ccRCC EC2 are enriched for functions related to focal adhesion. In addition, ccRCC EC2 exhibits a pronounced trajectory from normal epithelial cells to tumor cells (Figure S24A). These findings suggest a close association between endothelial cells and the process of tumor cell differentiation within ccRCC EC2.

Fibroblasts differentiate from BC EC4 to BC EC3, and finally to BC EC2 (Figure 6B). Ecotype BC EC2 comprises both tumor cells and fibroblasts. Notably, fibroblasts within BC EC2 are enriched for functions related to renal system development and MAP kinase activity, which may be associated with tumor progression.

In PCa, we observed that endothelium and fibroblasts differentiate from PCa EC2 to PCa EC3 (Figure 6C). Additionally, we found that tumor cells in PCa also undergo cross-ecotype differentiation, progressing from PCa EC3 to PCa EC2 and ultimately to PCa EC1 (Figure S24B). Fibroblasts in PCa EC3 were enriched for functions related to focal adhesion. Moreover, endothelial cells in PCa EC3 were enriched for the negative regulation of the execution phase of apoptosis (Table S6). These factors contribute to tumor progression.

Notably, the characteristic gene FOSL1 of ccRCC EC2 is also the TF that drives the differentiation of ccRCC tumor cells. Moreover, ccRCC EC2 and EC4 exhibited differential expression of previously identified high-risk genes influenced by methylation, CNVs and SNPs (Figure S24C). The characteristic gene ELF3 in tumor cells of PCa EC3 is also the TF driving the differentiation of PCa tumor cells. We further observed that the expression of high-risk genes—previously identified as being regulated by methylation, CNVs and SNPs—differs across PCa ecotypes. In addition, PCa tumor cells are inferred to follow a gradual transition from ecotype PCa EC3 to PCa EC2, and subsequently to PCa EC1. Notably, the expression levels of high-risk genes, including DLG5, SLC44A4 and HOXA10, show a decreasing trend across PCa EC3, PCa EC2 and PCa EC1 (Figure S24C). These findings suggest that the expression levels of risk genes influenced by genetic and epigenetic factors may be associated with tumor cell differentiation.

In addition, we found that distinct cancer types shared common functional changes during the process of cell differentiation. During CTL differentiation, the PI3K/AKT/mTOR pathway, G2/M checkpoint and DNA repair functions showed a downregulation of enrichment scores (Figure S20D,E). During macrophage differentiation, we observed a decrease in M1 signature scores accompanied by an increase in M2 scores. In addition, TNFA signaling, KRAS signaling, apoptosis, and immune regulatory pathway scores were reduced (Figure S21D). We further observed that macrophages exhibited increased tissue-residency features and the expression levels of the tissue-residency-associated gene FOLR2 and the polarization regulator TREM2 were upregulated (Figure S21E,F). Pseudotime analysis of endothelial cells revealed a differentiation trajectory from venous to arterial endothelial cells (Figure S22A–C). Additionally, this process was accompanied by upregulation of the Wnt/β-catenin pathway, which may contribute to therapy resistance in cancer (Figure S22D,E). During fibroblast differentiation, the Notch signaling pathway was upregulated during fibroblast differentiation (Figure S23C). These functional alterations in immune and stromal cells provided a supportive foundation for tumor cell progression (Figure 6E).

These results indicate that cell subpopulations are not confined to a single ecotype but exhibit differentiation trajectories that span across multiple ecotypes. Both genetic and epigenetic alterations contribute to the shaping of tumor ecosystems, whereby tumor cells, under these influences, modulate the functional states of other cellular subpopulations. These transitions reflect the plasticity of ecotypes, which can reshape cellular functions.

### 2.7. Screening Potential Therapeutic Agents Against Male Urologic Cancers

Targeted drugs suppress tumor progression by blocking key signaling pathways. In addition, an increasing number of studies have focused on TME-targeted therapies based on the differences between the TME and normal tissue environments [23]. Therefore, we aim to screen drugs separately based on tumor cell differentiation and ecotype characteristics.

**Figure 7:**
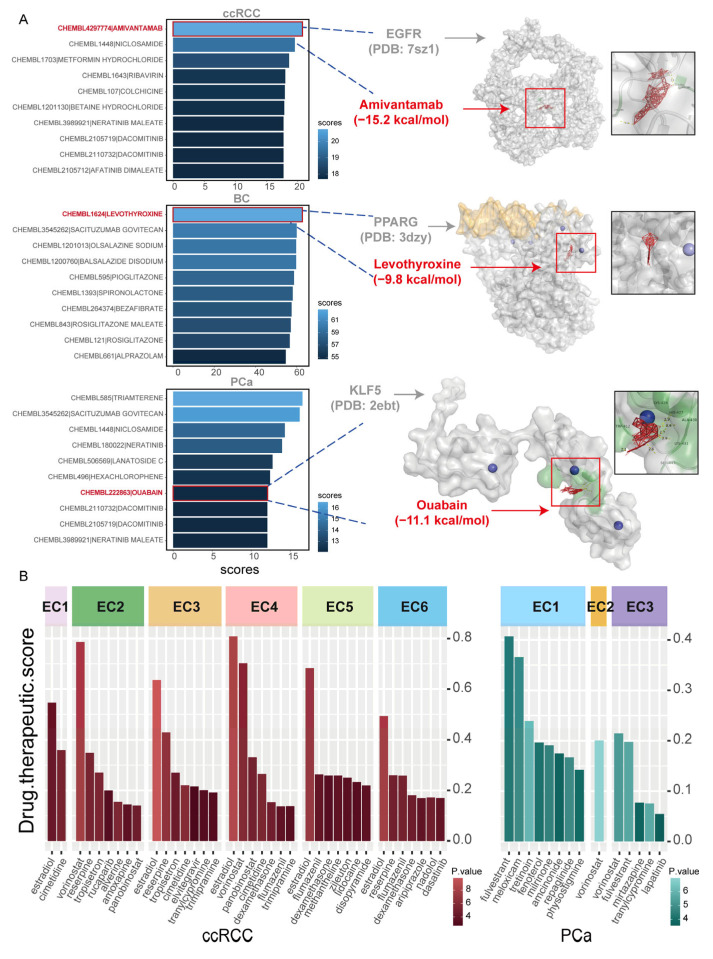
Therapeutic compound screening for male urological cancers. (**A**) (**Left**): Bar plot illustrating drug scores computed by Drug2Cell using CNV-driven gene regulatory networks in ccRCC and PCa, and CNV genes in BC. (**Right**): Molecular docking analysis of candidate drugs with their respective target genes. The red box indicates the region of interest for molecular docking. (**B**) Bar plot illustrating candidate drugs predicted by ASGARD through analysis of DEGs between cells in ecotypes and cells in normal tissues.

We used drug2cell to compare differences between normal epithelial cells and tumor cells, identifying significant candidate drugs. Based on the transcriptional regulatory networks of ccRCC and PCa, as well as the CNV genes during differentiation progression in BC, we further screened for candidate drugs (Figure 7A and Figure S24D). In this process, we discovered Amivantamab for ccRCC, which targets the EGFR gene; Levothyroxine for BC, which targets the PPARG gene; and Ouabain for PCa, which targets the KLF5 gene (Figure 7A and Table S7). We obtained several protein crystal structures corresponding to the target genes from the RCSB protein data bank (PDB) database. After importing both the target proteins and compound molecules into AutoDock Vina (ver 1.1.2), we calculated the affinity values of the best binding poses (Figure 7A). The binding affinities for all three drug-target pairs were ≤−9.00 kcal/mol, thus validating the therapeutic potential of these drugs at the molecular docking level.

In addition, we sought to identify targeted drugs based on these ecotype differences. We obtained scRNA data from normal kidney and prostate tissues and then applied the ASGARD algorithm to evaluate the efficacy of drugs from the LINCS L1000 database (Figure S24E,F). Through this analysis, we identified several potential therapeutic drugs specific to different ecotypes (Figure 7B). For instance, high scores of vorinostat were observed in ccRCC EC2 and ccRCC EC4, both of which contain tumor cells. Meanwhile, reserpine exhibited a relatively high drug score in ccRCC EC2. In addition, fulvestrant was identified as a candidate drug for PCa EC1, while vorinostat emerged as a potential candidate for PCa EC2 and PCa EC3.

By identifying drug targets during tumor cell differentiation, we screened for drugs influencing tumor cell differentiation. Additionally, we evaluated the effects of drugs on ecotypes. These results may provide a foundation for future studies addressing acquired resistance and drug combinations.

## 3. Discussion

Male urological cancers present complex clinical characteristics that pose significant therapeutic and prognostic challenges. The immune heterogeneity of ccRCC remains incompletely characterized [24]. In BC, tumor cell heterogeneity is closely associated with tumor progression [25]. For PCa, the Gleason score system [26], and higher Gleason scores are strongly associated with increased tumor aggressiveness and poorer clinical outcomes [27]. In this study, we first constructed a multi-omics atlas of male urological cancers. We then investigated the molecular mechanisms by which CNVs, DNA methylation, and SNPs regulate gene expression during tumor cell differentiation. Subsequently, we analyzed tumor cell interactions and examined how cross-ecotype differentiation influences ecotype composition and function. Finally, we screened for potential therapeutic drugs targeting the molecular mechanisms of tumor cell differentiation and ecotypes containing tumor cells.

Based on multi-omics data, we constructed a comprehensive multi-omics atlas of male urological cancers. We identified tumor-specific and shared TFs. We observed significant enrichment of the FOSL1 motif in ccRCC epithelial cells, consistent with previous reports showing that FOSL1 promotes metastasis in ccRCC [28]. Similarly, the FOXA1 motif was enriched in PCa epithelial cells, where FOXA1 facilitates androgen receptor (AR) signaling and supports prostate tumor growth and survival [29]. Epithelial cells in both ccRCC and PCa displayed enrichment of the TFs NFIB and NFIA, whereas endothelial cells were characterized by NFKB1 enrichment. Moreover, SPIC and SPI1 activities were consistently elevated in myeloid cells across all three cancer types. Additionally, spatial transcriptomic data show that epithelial cells in PCa are highly spatially associated with stromal cells, and epithelial cells in ccRCC are highly associated with lymphoid cells, emphasizing the need for deeper investigation into the TME of these cancers.

Tumor cells in male urological cancers generally originate from epithelial cells [30]. Using CNV analysis, we identified normal epithelial cells and tumor cells, as well as subclones within the tumor cells. Additionally, we identified CNV-associated genes. Several previously reported mutation-prone genes were detected in our analysis [31,32,33]. Through the analysis of CNVs in tumor cells, we identified both commonalities and specific features across different male urological cancers. We further assessed the functional impact of these copy number alterations within the subclone. In addition, three cancer types shared copy number losses on chromosome 6, suggesting a potential link to the immune response. Analysis of sex chromosome CNVs revealed copy number gains on chromosome X across three cancers, which may affect protein ubiquitination-related pathways.

Tumor cells exhibited tumor-specific marker expression, such as FOLH1 and KLK3 in PCa and CA9 in ccRCC [34,35,36]. Therefore, we constructed pseudotime trajectories separately for each cancer type. We found that tumor cell differentiation is associated with tumor progression and is accompanied by a significantly attenuated interferon response. We then identified TFs driving differentiation and constructed TF regulatory networks (ccRCC: FOSL1, RUNX1, NFIB, NFIC, CEBPB, HNF4A, ID4; PCa: ETS2, KLF10, CEBPB, ELF3, TEAD1, KLF5, FOS, FOSB). Further analysis showed that the expression levels of the CNV gene S100A6, regulated by KLF5, and the CNV gene COX6A1, regulated by NFIC, ID4, HNF4A and NFIB, both changed as tumor cell differentiation. Previous studies have reported that NFIC and ID4 influence the development and progression of tumor cells [37,38]. The loss of S100A6 protein expression is common in PCa development [39]. These results suggest that TF regulatory networks may influence tumor progression.

To further explore the deeper regulatory mechanisms of gene expression during tumor cell differentiation, we incorporated data from other omics layers to conduct a more comprehensive, multidimensional analysis. DNA hypomethylation has been linked to the onset and progression of cancers [40,41]. Among the differentially methylated genes, the gene set BEN-PORATH_ES_WITH_H3K27ME3, which may influence cell differentiation, showed the highest enrichment [42]. Integrative analysis incorporates CNV region, methylation profiling and SNP data, revealing novel mechanistic insights. Through comprehensive genomic characterization, we further delineate how multiple genetic alterations converge to modulate risk genes such as FLI1 and TRIM31. The peaks linked to the risk genes are influenced by methylation, CNV and SNP, which inversely regulate gene expression. This analysis focused on the impact of DMR on peaks along pseudotime trajectories. This approach may not fully reflect the regulatory relationship between dynamic changes in methylation and chromatin accessibility during pseudotime trajectories. Our analysis of the complex regulatory mechanisms affecting these genes provides a more comprehensive and detailed understanding than examining gene changes from a single perspective.

Tumor progression is profoundly influenced by the interactions between tumor and their environment, interactions that ultimately determine whether the primary tumor can be eradicated, metastasize, or develop into dormant micrometastases [43]. It is believed that the TME is not merely a silent bystander but an active facilitator in tumor progression. Based on gene expression, cells can be classified into distinct cell modules, which can serve as ecotypes for further investigation. We employed Ecotyper to define ecotypes in male urological cancers. Ecotype clustering was predominantly driven by cells spanning the major lineages. Among these ecotypes, fibroblasts and endothelial cells may contribute to tumor progression. In addition, CNVs may influence the functional states of tumor cells within ecotypes as well as their interactions with other cell types. Such tumor cells promote tumor growth and progression by interacting through the ligand EFNA1 with the receptor EPHA3 expressed on myofibroblasts [44]. In BC EC2, myofibroblasts are enriched for functions related to renal system development and MAP kinase activity, which may further promote tumor progression through the NRG1-ERBB3 signaling pathway [45,46]. In the ecotypes of PCa and ccRCC, tumor cells interacted with macrophages through the APP-CD74 axis, which may influence macrophage function [47].

In the pseudotime analysis, we examined the differentiation trajectories of cell types across ecotypes within each cancer. Cellular differentiation is not confined within a single ecotype but rather exhibits cross-ecotype features, with cellular functions being modulated by interactions with other cell types. Previously identified TFs and risk genes may influence the differentiation of tumor cells within ecotypes. Furthermore, we identified shared functional changes occurring during cell differentiation of the same cell types across all three cancers. During CTL differentiation, the PI3K/AKT/mTOR and mTORC1 pathways were downregulated, both of which are associated with T cell growth [48,49,50]. Notably, macrophages exhibited increased M2 and tissue-residency signatures, suggesting a potential role in modulating the surrounding TME [51,52,53]. Pathway enrichment changes during stromal cell differentiation (Wnt/β-catenin pathway in endothelial cells and Notch signaling pathway in fibroblasts) may contribute to tumor progression.

Finally, we screened potential therapeutic agents targeting the molecular mechanisms underlying tumor cell differentiation (Amivantamab in ccRCC, Levothyroxine in BC, Ouabain in PCa). We identified drugs targeting the ecotypes. For instance, vorinostat targets ccRCC EC2 and ccRCC EC4, while fulvestrant targets PCa EC1. Reserpine has been reported to induce acute kidney injury [54]; we observed that it also possesses potential anti-tumor effects in ccRCC EC2. These findings indicate that suitable therapeutic drugs for patients can be identified from multiple perspectives, enabling more precise and personalized treatment strategies.

In summary, our study presents a high-resolution transcriptomic, chromatin accessibility, and spatial landscapes of male urological cancers. Our research examines the roles of various cell subpopulations in male urological cancers, providing a comprehensive view of gene expression and regulation in male urological cancers. And we also uncover mechanisms underlying ecotypes in different cancers. These findings offer new perspectives on understanding the malignant progression of male urological cancers and provide valuable insights for developing novel therapeutic strategies for male urological cancers.

## 4. Materials and Methods

### 4.1. Sample Information

The dataset used in this study is publicly available from the NCBI Gene Expression Omnibus (GEO; https://www.ncbi.nlm.nih.gov/geo/, accessed on 16 January 2025). The accession number for BC scRNA-seq is GSE222315 [55], and those for PCa scRNA-seq are GSE176031 [56], GSE193337 [57] and GSE157703 [58]. The accession number for ccRCC scRNA-seq and scATAC-seq samples is GSE207493 [59]. The accession number for PCa spatial transcriptomics samples is GSE207493 [60]. The PCa scATAC-seq dataset used in this study is publicly available from the NCBI (https://www.ncbi.nlm.nih.gov/Traces/study/, accessed on 16 January 2025) under accession number PRJNA720090 [61]. The ccRCC spatial transcriptomics dataset used in this study is publicly available from the Biostudies (https://www.ebi.ac.uk/biostudies, accessed on 16 January 2025) under accession number E-MTAB-12767 [62]. Detailed sample accession information is provided in Table S1. The TCGA transcriptomic and DNA methylation data for the three cancers were obtained from the UCSC Xena Browser (https://xenabrowser.net/datapages/, accessed on 16 January 2025). The TCGA CNV and SNP data were downloaded from the TCGA database using the TCGAbiolinks package. The accession number for ccRCC normal adjacent scRNA-seq is GSE20749359, for BC validation scRNA-seq is GSE267718 [63], and those for PCa scRNA-seq are GSE176031 [56] and GSE193337 [57]. This paper analyzes existing, publicly available data. The datasets generated during and/or analyzed during the current study are available from the corresponding author upon reasonable request.

### 4.2. Cell Type Annotation in scRNA-seq

First, scRNA-seq data were preprocessed using the R package Seurat (ver 4.4.0), and low-quality cells were filtered out [nFeature_RNA > 200 & nFeature_RNA < 7500 & percent.mt < 10% & nCount_RNA > 300 & percent. HB < 5%] [64]. Potential doublets were detected and filtered out using the R package DoubletFinder (ver 2.0.3). Principal components (PCs) were then computed using the RunPCA function with 3000 variable genes, and the significant PCs (PCs with cumulative contribution greater than 90%, individual PC contribution to variance less than 5%, or difference between consecutive PCs less than 0.1%) were calculated. Subsequently, we applied batch correction using the RunHarmony function in Harmony (ver 1.2.0) with lambda = 1. Following the correction, the dataset was reprocessed for downstream analyses, including dimensionality reduction and clustering. We performed clustering using the FindNeighbors and FindClusters functions (resolution = c (1:10/10)) to identify distinct cell clusters. We applied the RunTSNE and RunUMAP functions to perform dimensionality reduction on the processed dataset. Cell types were assigned to each cluster by examining the expression of canonical marker genes.

### 4.3. xCell Analysis

TPM-normalized gene expression data for PCa and male ccRCC patients were obtained from the UCSC Xena Browser (https://xenabrowser.net/datapages/, accessed on 5 November 2025). Immune cell infiltration across distinct pathological subgroups was assessed using the xCell (ver 1.0.0) [65]. Boxplots of immune scores across different pathological groups were generated using ggplot2 (ver 3.5.1).

### 4.4. TF Activity Analysis of scRNA-seq

TF activity at the single-cell level was further estimated using the R package decoupleR (ver 2.9.7) [66,67]. The CollecTRI gene regulatory network was obtained using the get_collectri function. Expression profiles from the scRNA-seq datasets of the three cancer types were then used as input for the run_ulm function to infer TF activity networks. DecoupleR was used to infer TF activity from scRNA-seq data. We identified TFs with high activity in the same cell types across the three cancers. We then examined TF-associated motifs based on motif enrichment results from marker peaks identified in the scATAC-seq analysis to determine TFs shared by corresponding cell types across the three cancers. The results from decoupleR and motif are provided in Table S1.

### 4.5. Spatial Transcriptome Analysis

For the 10× Genomics ccRCC spatial transcriptomics data, normalization was performed using the SCTransform function in Seurat. Cell clustering was then conducted with FindNeighbors and FindClusters (dims = 1:30), followed by dimensionality reduction using RunUMAP (dims = 1:30). For the Slide-seq PCa spatial transcriptomics data, normalization was similarly performed with SCTransform (ncells = 3000), followed by clustering with FindNeighbors and FindClusters (dims = 1:20) and dimensionality reduction using RunUMAP (dims = 1:20). Subsequently, scRNA-seq data from PCa samples were used as a reference, while 10% of cells from each cell type in the ccRCC single-cell dataset were randomly selected as reference subsets. Anchors between reference and query datasets were identified using FindTransferAnchors (normalization.method = “SCT”; npcs = 50 for Slide-seq data). Cell type compositions for spatial transcriptomics spots were then predicted using TransferData, with dimensions set to 1:50 for Slide-seq and 1:30 for ccRCC. Subsequently, we applied MISTy (ver 1.14.0) (Multiview Intercellular SpaTial modeling framework) to systematically analyze spatial interaction networks among distinct cell types in ccRCC and PCa separately [68]. CCI networks were visualized using the plot_interaction_communities function to depict relationships between different cell types.

### 4.6. CNV Analysis

CNVs in epithelial cells were detected using the inferCNV (ver 1.20.0 cutoff = 0.1, denoise = TRUE, HMM = TRUE) of the Trinity CTAT Project. Lymphoid cells were used as the baseline for assessing CNV in epithelial cells. The inferred CNV on each chromosome was visualized in different cancers using the R pheatmap function. Epithelial cells clustering together with controls were annotated as Normal epithelial cells, whereas the remaining cells were annotated as Tumor cells. The tumor cells were then run with inferCNV again to infer CNVs. We identified the existence of distinct CNVs within tumor cells and defined tumor cell subpopulations harboring distinct CNVs as subclones [69]. We then identified differentially expressed genes (DEGs) between subclones and normal epithelial cells. Through integrative analysis, we selected genes with consistent fold change (FC) in expression and CNV (copy number gain or loss) as CNV genes for further analysis. For each subclone, we first performed functional enrichment analysis based on its CNV genes. Next, CNV genes on each chromosome were categorized as gain or loss, ranked by their fold change values, and then subjected to gene set enrichment analysis (GSEA) based on Gene Ontology (GO), Kyoto Encyclopedia of Genes and Genomes (KEGG) and Hallmark gene sets [70,71,72,73]. To investigate CNVs on sex chromosomes, tumor subclones were additionally analyzed with inferCNV, applying the chr_exclude parameter to specifically infer CNVs on chromosomes X and Y. For CNV analysis of bulk RNA data, CNV data were downloaded from TCGA using TCGAbiolinks. In both the ccRCC and BC cohorts, only samples annotated as male in the phenotype data were retained for subsequent analyses. GISTIC2.0 analysis was subsequently performed using the GenePattern platform [74] (https://cloud.genepattern.org/gp/pages/login.jsf, accessed on 5 November 2025).

### 4.7. Pseudotime Trajectory Analysis of scRNA-seq

We employed Monocle3 (ver 1.3.7) to infer pseudotime trajectories from scRNA-seq data. In the differentiation trajectory analysis of epithelial cells, normal epithelial cells were chosen as the root. We performed pseudotime trajectory analysis on BC samples and on ccRCC and PCa samples with pathological subgroups information. Batch effects were corrected using the align_cds function with the alignment_group parameter. For immune cells and stromal cells, we used CytoTRACE2 (ver 1.1.0) to predict their differentiation potential and selected the cell cluster with consistently high scores across male urological cancers as the root [75]. We then inferred the transition of cells from one state to another and identified CNV-associated genes whose expression levels changed during cell type differentiation [76].

### 4.8. Pseudotime-Related Functional Enrichment Analysis

Genes differentially expressed along the single-cell trajectory were identified using graph_test. Genes with Moran’s I > 0.25 were selected for further analysis. For each selected gene, expression values along the pseudotime trajectory were extracted from the Monocle3 object and ordered according to the pseudotime of each cell. To reduce noise and capture dynamic trends, gene expression profiles were smoothed using the smooth.spline function with three degrees of freedom. The resulting smoothed expression values were subsequently standardized by subtracting the mean and dividing by the standard deviation, yielding z-scores. Genes were clustered based on z-scores using the K-means algorithm, resulting in distinct gene clusters. Functional enrichment analysis for each gene cluster was performed using the R package clusterProfiler (ver 4.12.2) [77]. We plotted the significantly enriched pathways [p_adj value less than 0.05] as bar charts. Additionally, we used the AddModuleScore function in Seurat to score each cell based on Hallmark gene sets. Cells were ordered according to pseudotime, and the trajectory of gene set scores along pseudotime was visualized using the geom_smooth function to generate fitted curves.

### 4.9. Cell Annotation and Pseudotime Analysis of scATAC-seq Data

For the PCa dataset, we downloaded the SRA files from the SRA database and converted them to FastQ format using the SRA toolkit. The reads were aligned to the human reference genome (hg38) using BWA. For single-end FastQ sequencing data, we performed alignment with snaptools align-single-end; for paired-end FastQ data, snaptools align-paired-end was used to generate SAM files. Subsequently, SAM files were converted to BAM format using samtools, followed by PCR duplicate removal with sambamba. Fragment information was then extracted from the BAM files to create fragment files. Next, we imported the fragment files into ArchR (ver 1.0.3) (https://github.com/GreenleafLab/ArchR, accessed on 20 March 2025). After preprocessing, we applied the iterative latent semantic indexing (LSI) method to reduce the dimensionality of the sparse insertion counts matrix from thousands to tens or hundreds. Batch effects were corrected by sample using the addHarmony function in ArchR with default parameters. Canonical correlation analysis (CCA) was then used to match scRNA-seq and scATAC-seq data [78], with default parameters. We used ArchR to identify the cell types of each cell cluster in the scATAC data using a label transfer method. Subsequently, cells were clustered using the addClusters function in ArchR, and the results were visualized using UMAP. We used the addTrajectory function to assign trajectories to the corresponding cells. Along the pseudotime trajectory, TFs influencing tumor cell differentiation were identified based on motif enrichment, gene scores, and gene expression patterns. By integrating peak-to-gene links with CNV genes exhibiting dynamic expression changes in the pseudotime analysis of scRNA-seq data, we obtained CNV genes linked to peaks during differentiation. Peaks containing the motifs were then selected to construct a TF regulatory network driving tumor cell differentiation through the regulation of CNV genes.

### 4.10. Methylation Analysis

We obtained the TCGA 450K DNA methylation array data and corresponding phenotype data from the UCSC Xena Browser (https://xenabrowser.net/datapages/, accessed on 10 March 2025). In both the ccRCC and BC cohorts, only samples annotated as male in the phenotype data were retained for subsequent analyses. DMRs between tumor and normal tissue samples were identified using the champ.DMR function in the R package ChAMP (ver 2.34.0) with default parameters [79]. Functional enrichment analysis of the DMRs was then performed using the champ.GSEA function, with an adjusted *p*-value cutoff of 0.05. Additionally, DMRs on chromosomes X and Y were retained by applying the champ.filter (betaData, pd = pd, filterXY = FALSE), and these DMRs were subsequently overlapped with chromatin accessibility peaks for genetic and epigenetic analysis.

### 4.11. Genetic and Epigenetic Analysis

Chromatin accessibility peaks showing dynamic changes during tumor cell differentiation were identified in ccRCC and PCa. DMRs were overlapped with these peaks using the findOverlaps function from the GenomicRanges R package. Similarly, CNV regions obtained from the inferCNV results of scRNA were overlapped with the peak set. Peaks that overlapped with both DMRs and CNV regions were then compiled, representing candidate regulatory elements potentially modulated by multiple layers. Methylated regions were annotated using the GRanges function. The plotBrowserTrack function, with the parameter highlight = highlight_GRanges, was subsequently employed to visualize the methylated regions, including their overlap with peaks and genes associated with these peaks.

### 4.12. SNP Analysis

SNP data for male patients were retrieved from TCGA using the TCGAbiolinks (ver 2.34.0) R package, and genes harboring somatic mutations were identified [80]. Regulatory target genes associated with the previously defined chromatin accessibility peaks were determined using the peak2gene function. The chromosomal locations of SNPs occurring within these genes were then annotated using the plotBrowserTrack and GRanges function, enabling integration of mutation data with chromatin accessibility-linked regulatory elements.

### 4.13. Ecotype Analysis

EcoTyper employs a community detection algorithm to identify robust networks of co-associated cell states, termed ecotypes [15,81]. We applied EcoTyper separately to BC tumor samples and to ccRCC and PCa tumor samples with pathological grading information in order to infer cell states and tumor ecotypes. The analysis used count matrices, cell type annotations, and sample information from scRNA-seq data as input, with default parameters. We identified ecotypes by Ecotyper in male urological cancers. The feature genes of the cell states comprising each ecotype are listed in Supplementary Table S5. Feature genes for each cell state, as provided in the gene_info.txt files generated by EcoTyper, were subjected to functional enrichment analysis using the clusterProfiler R package to elucidate the biological processes associated with each cell state [82].

### 4.14. Cell–Cell Communication in Ecotypes

Cell subpopulations within each ecotype were identified based on the identification of cellular subpopulations in scRNA-seq. For each ecotype, cell–cell interactions (CCIs) were predicted using CellPhoneDB (ver 5.0.0) by leveraging known ligand-receptor pairs [83]. Statistically significant ligands and receptors associated with “sender” and “receiver” cell populations were rigorously identified through CellPhoneDB’s permutation-based analysis.

### 4.15. Spatial Distribution of Ecotypes

To spatially resolve the distribution of ecotypes, we performed clustering of all spatial transcriptomics spots using seurat separately for ccRCC and PCa to identify distinct spot clusters, referred to as CCs. Gene Set Variation Analysis (GSVA) was then conducted by the R package GSVA (ver 1.52.3) on each cluster to calculate enrichment scores for GO, KEGG, and Hallmark gene sets, allowing for functional annotation of the spatially defined regions [84]. Ecotype-specific signatures—derived from characteristic gene expression patterns of cellular states in scRNA-seq data—were then projected onto spots using the AddModuleScore. This approach enabled quantitative assessment of ecotype enrichment patterns across tissue architectures, revealing spatially restricted niches of ecotype activity.

### 4.16. Drug Analysis

Based on scRNA-seq data of tumor cells and normal epithelial cells, we applied Drug2Cell (ver 0.1.2) (https://github.com/Teichlab/drug2cell, accessed on 14 June 2025) to identify candidate drugs and their corresponding target genes [85]. To achieve a more accurate identification of candidate drugs, target genes were selected from two critical sources: (1) the TF–CNV gene regulatory networks implicated in driving tumor cell differentiation in PCa and ccRCC; (2) CNV genes that exhibit differential expression during tumor cell differentiation in BC. As a result, we obtained candidate drugs that may modulate the differentiation of tumor cells. To further assess drug–target interactions, we conducted molecular docking and visualized the results using AutoDock Vina [86]. In addition, scRNA-seq data from normal tissues were used as a reference to identify cell-type-specific DEGs within each ecotype. These gene signatures were subsequently used as input for ASGARD (ver 1.0.0) to predict potential drugs targeting each ecotype [87].

## 5. Conclusions

In summary, this study systematically dissects the shared and distinct features of male urological cancer ecotypes from a multi-omics perspective. We constructed a comprehensive multi-omics atlas of male urological cancers and revealed the important role of epithelial cells in tumor progression. We further characterized cancer ecotypes and analyzed the functional properties of tumor subclones and their interactions with other cell types. By integrating cross-ecotype differentiation trajectories, we identified key transcription factors, including FOSL1 and KLF5, that drive tumor cell differentiation, and further elucidated the genetic and epigenetic mechanisms governing this process. Finally, we identified potential therapeutic agents targeting tumor cell differentiation and ecotype-specific signature genes. These findings provide new insights into the tumor heterogeneity of male urological cancers and offer valuable guidance for precision medicine strategies. 

## Data Availability

The raw data supporting the conclusions of this article will be made available by the authors upon request. The R codes used for our study are available at https://github.com/yuhe-code/Male-urological-cancers, accessed on 17 November 2025.

## Supplementary Materials

The following supporting information can be downloaded at https://www.mdpi.com/article/10.3390/ijms27062712/s1.

## Abbreviations

The following abbreviations are used in this manuscript:

AR	Androgen receptor
BC	Bladder cancer
ccRCC	Clear cell renal cell carcinoma
CNV	Copy number variation
CC	Compositional cluster
CMR	CNV-associated methylation region
CTL	Cytotoxic T lymphocyte
CCA	Canonical correlation analysis
CCI	Cell–cell interaction
DEG	Differentially expressed gene
DMR	Differentially methylated region
EMT	Epithelial–mesenchymal transition
GSVA	Gene set variation analysis
GSEA	Gene set enrichment analysis
GEO	Gene expression omnibus
GO	Gene ontology
HG	High Gleason score group
LG	Low Gleason score group
LSI	Latent semantic indexing
KEGG	Kyoto encyclopedia of genes and genomes
NMF	Non-negative matrix factorization
MP	Meta-program
PDB	RCSB protein data bank
PC	Principal components
PCa	Prostate cancer
PPI	Protein–protein interaction
SNP	Single-nucleotide polymorphism
TCGA	The cancer genome atlas
TF	Transcription factor
TME	Tumor microenvironment
