# Supplementary material for: Integrating Multi-Omics Atlas to Uncover Genetic and Epigenetic Mechanisms and Reveal Cell State Evolution Across Ecotypes in Male Urological Cancers
Source: Int J Mol Sci. 2026 Mar 16;27(6):2712. doi: 10.3390/ijms27062712 (PMC13026699; doi:10.3390/ijms27062712)
Supplement: Supplementary file 1 [file ijms-27-02712-s001.zip › Supplementary Figure.pdf]

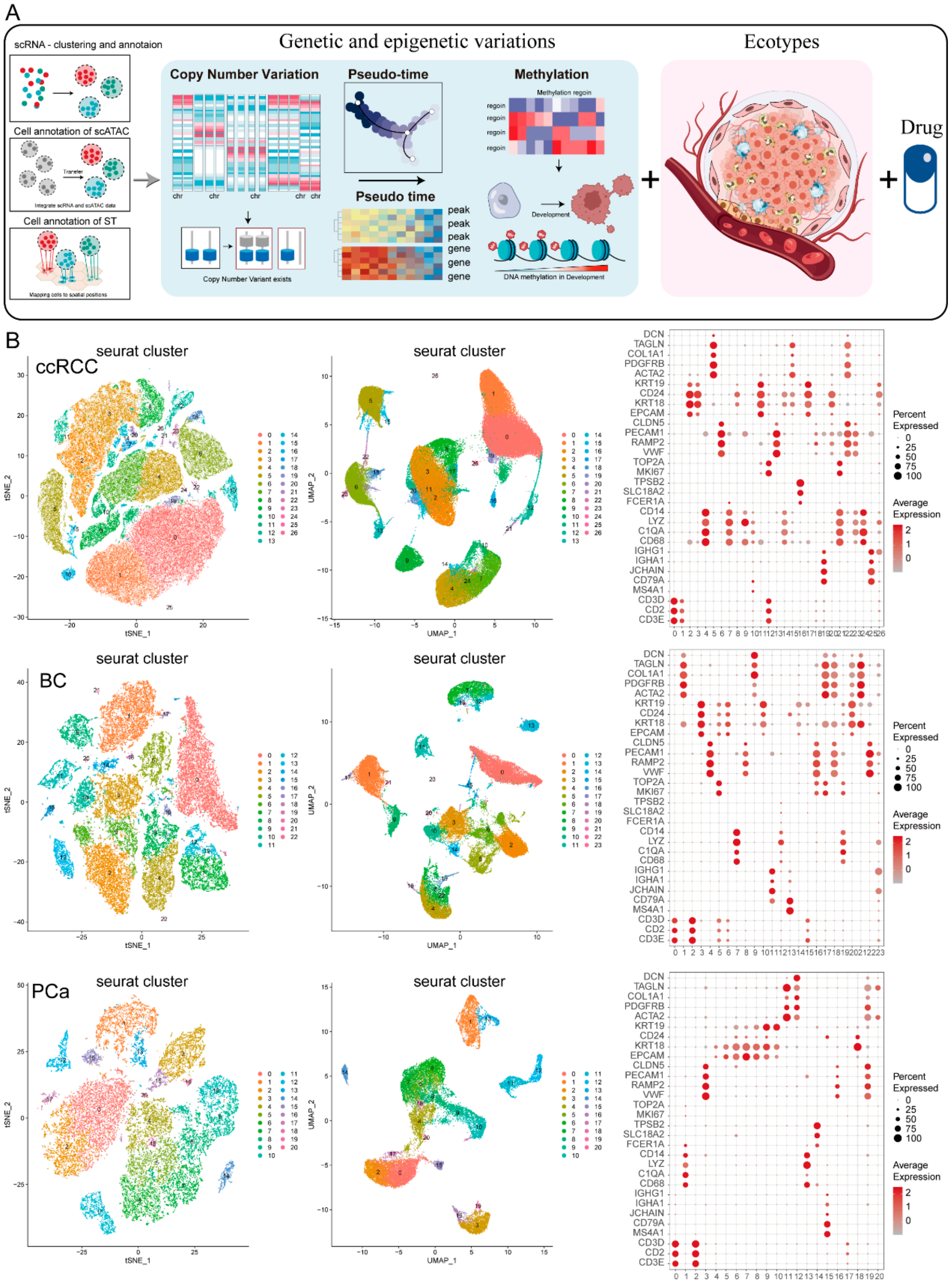

Figure S1. Workflow and clustering of single-cell RNA-seq data.

(A) Schematic overview of the study design and analytical workflow. (B) Dot plots showing marker gene expression in cell clusters across scRNA-seq data from different cancers. t-SNE and UMAP plots depict dimensionality reduction of the same datasets.

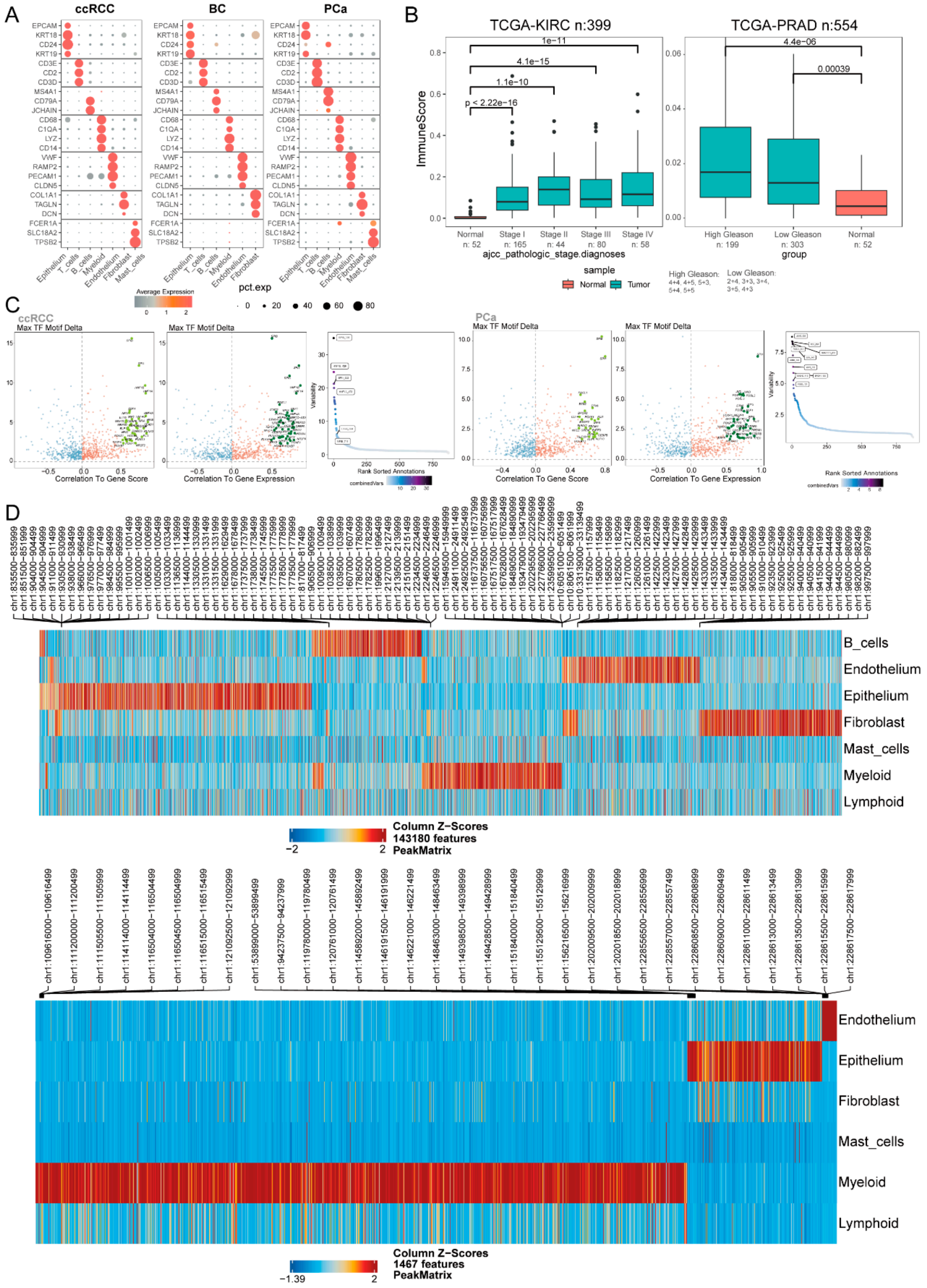

Figure S2. Cell annotation of scRNA-seq data and identification of cell-type–specific peaks and motifs.

(A) Dot plot showing the expression of marker genes across different cell types. (B)

ImmuneScores of TCGA data were calculated using xCell. (C) Gene expression, functional scores, and ranking of TF. (D) Heatmap showing the marker peaks of cell type.

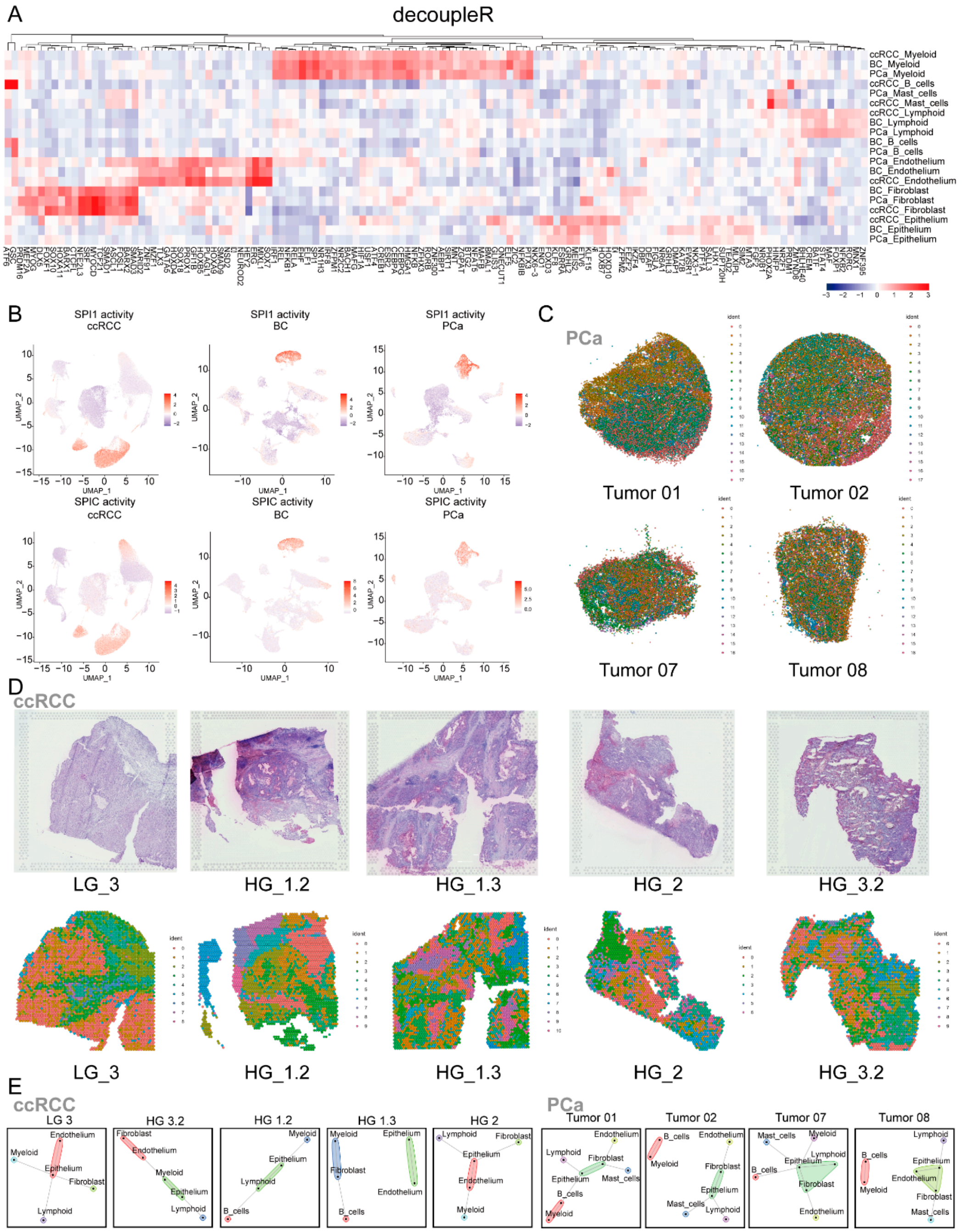

Figure S3. Identification of TFs and analysis of spatial transcriptomics data.

(A) The mean value of TFs activity in different cell types was inferred using decoupleR.

(B) Feature plots showing the activity of transcription factors SPIC and SPI1 in cells. (C) Spatial distribution of spot clusters for each PCa sample. (D) Spatial distribution of spot clusters for each ccRCC sample and corresponding tissue sections. (E) Dependencies between cell types across different samples.

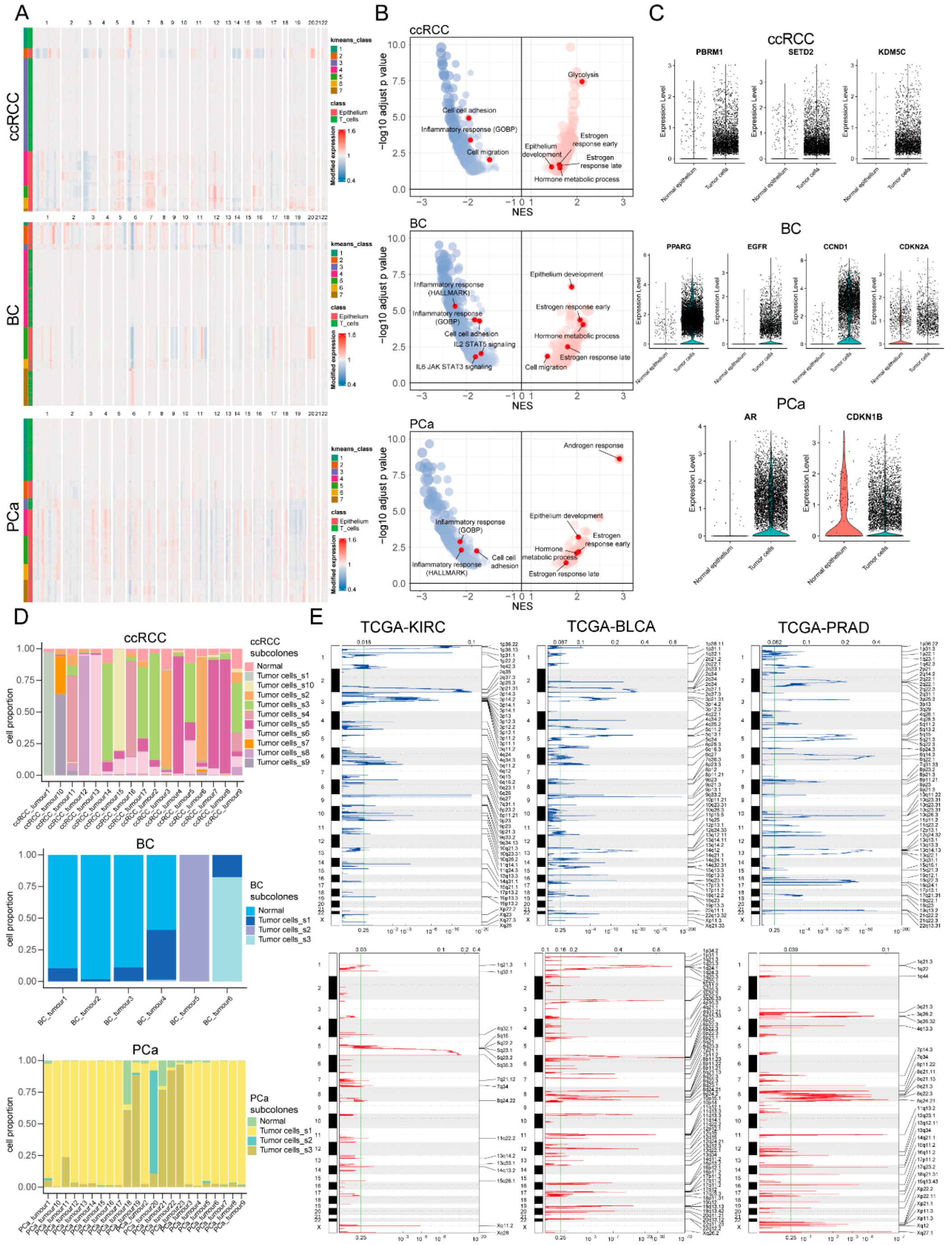

Figure S4. CNV landscape of male urological cancers.

(A) Heatmap showing CNVs in epithelial cells and T cells. (B) Volcano plot showing the GSEA of CNV genes differentially expressed between tumor and normal epithelial cells. (C) Violin plots showing the expression of CNV genes in normal epithelial and tumor cells. (D) Proportions of tumor subclones across different samples. (E) GISTIC2.0 analysis of CNV data of male sample from the TCGA database.

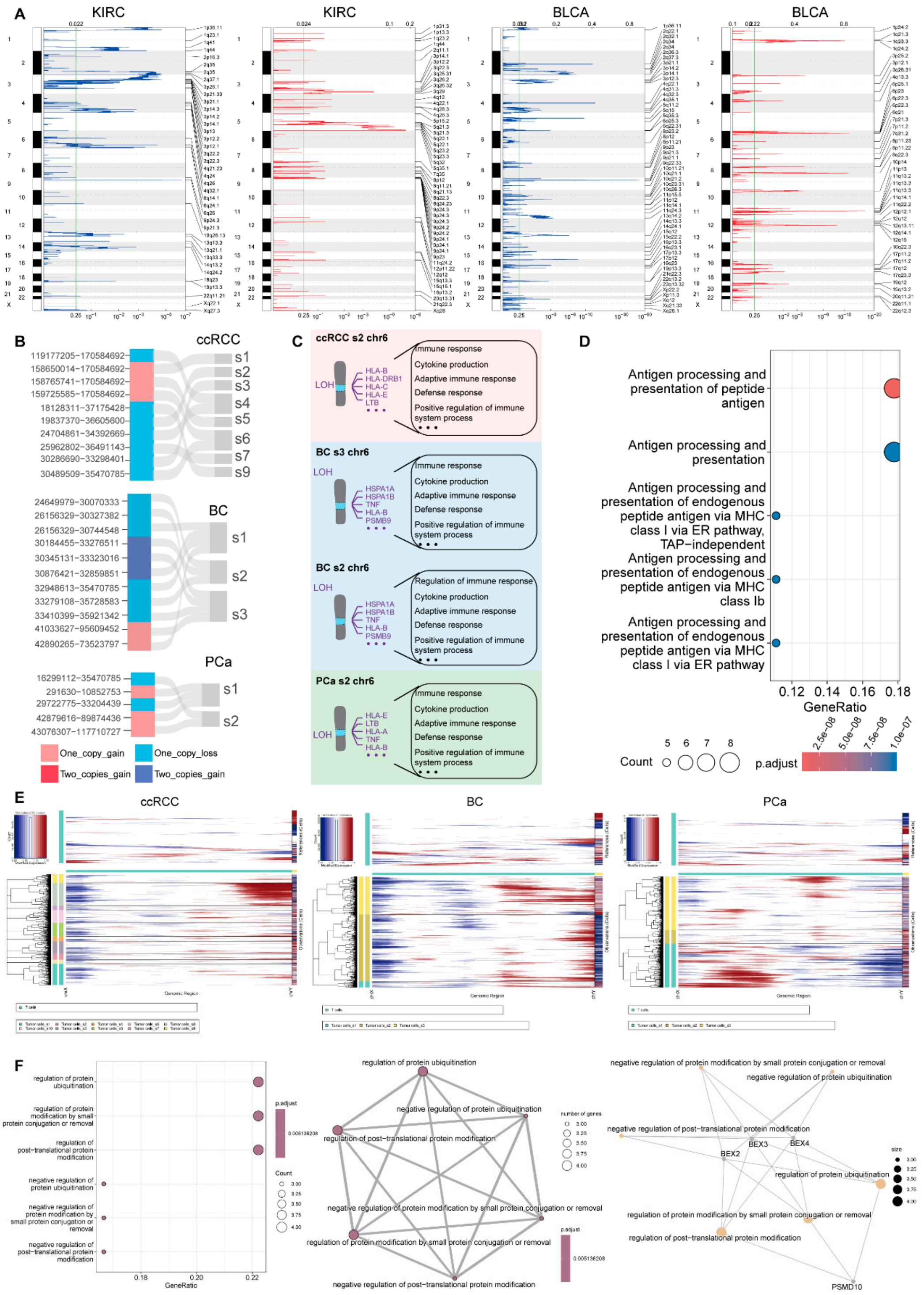

Figure S5. CNV landscape of male urological cancers.

(A) GISTIC2.0 analysis of CNV data of female sample from the TCGA database. (B) Sankey plot illustrating regions of amplification and deletion on chromosome 6 in

various tumor types. (C) Functional enrichment analysis of genes affected by CNV deletions on chromosome 6 in tumor cell subclones. (D) Dot plot showing functional enrichment analysis of genes on chromosome 6 with copy number loss across three cancer types. (E) InferCNV analysis of chrX and chrY across different tumor cell subclones. (F) Dot plot showing functional enrichment analysis of genes with copy number amplification on chromosome X. Networks shoing pathway–pathway and pathway–gene relationships.

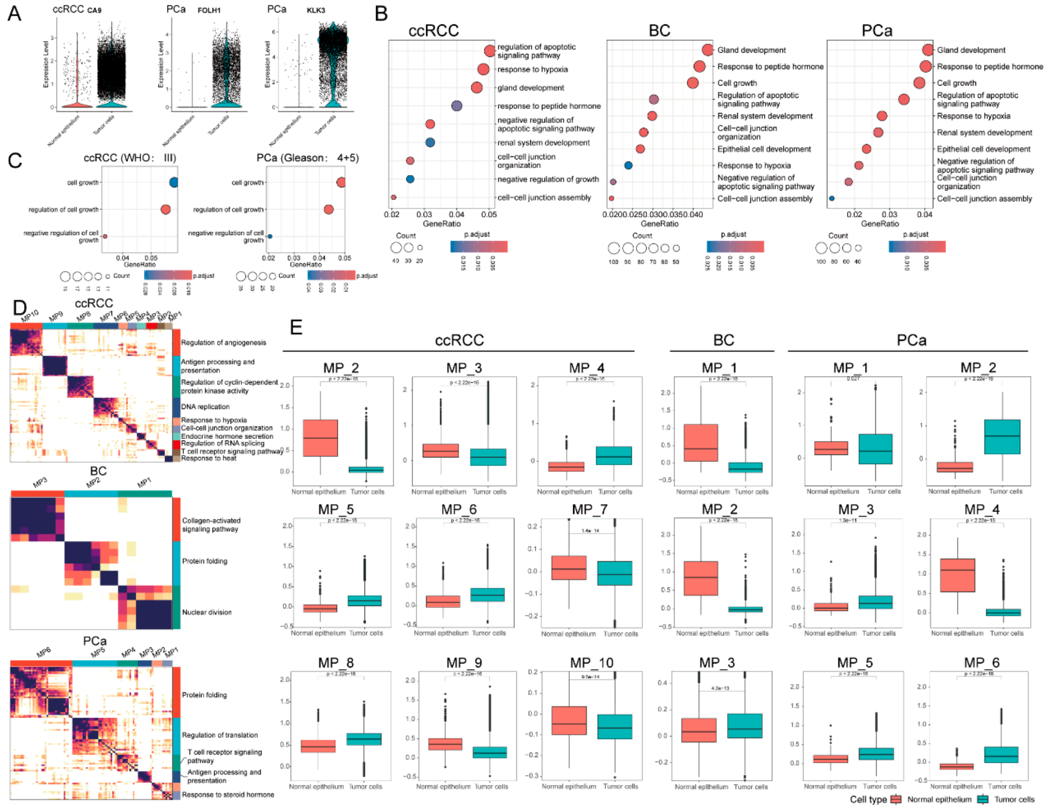

Figure S6. Identification of gene modules based on NMF analysis of epithelial cells. (A) Expression of tumor-specific markers in epithelial cells. (B) Common functional enrichment of DEGs between tumor and normal cells in three cancer types. (B) Functional enrichment analysis of DEGs from tumor cells across different pathological groups. (D) Gene modules identified by NMF analysis of epithelial cells, along with

functional annotation of each module. (E) Gene module enrichment scores in tumor and normal epithelial cells shown as box plots.

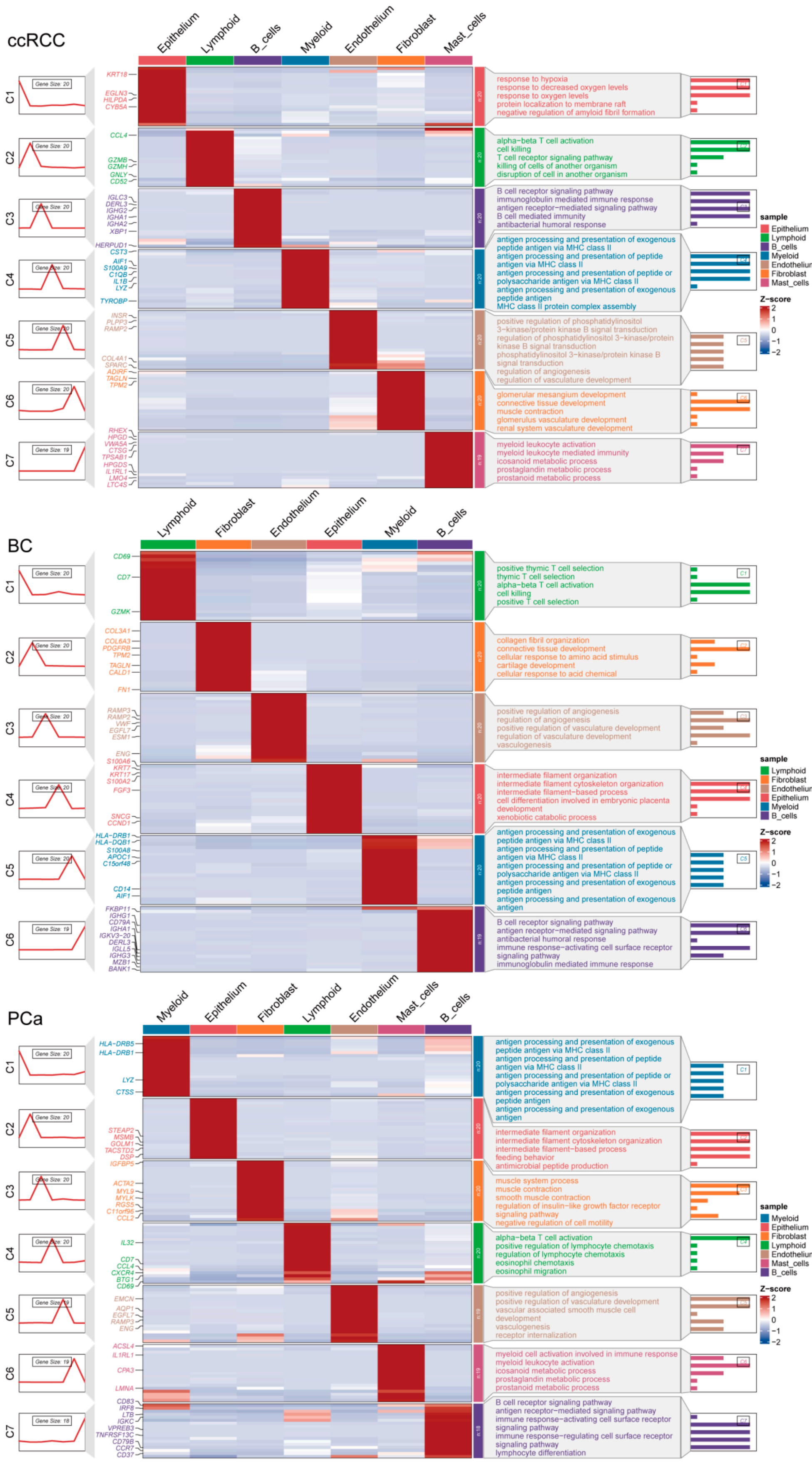

ccRCC
Epithelium
Lymphoid
B_cells
Myeloid
Endothelium
Fibroblast
Mast_cells
C1
C2
C3
C4
C5
C6
C7
Gene Size: 20
Gene Size: 19
KRT18
EGLN3
HILPDA
CYB5A
CCL4
GZMB
GZMH
GNLY
CD52
IGLC3
DERL3
IGHG2
IGHA1
IGHA2
XBP1
HERPUD1
CST3
AIF1
S100A9
C1QB
IL1B
LYZ
TYROBP
INSR
PLPP3
RAMP2
COL4A1
SPARC
ADIRF
TAGLN
TPM2
RHEX
HPGD
VWA5A
CTSG
TPSAB1
HPGDS
IL1RL1
LMO4
LTC4S
response to hypoxia
response to decreased oxygen levels
response to oxygen levels
protein localization to membrane raft
negative regulation of amyloid fibril formation
alpha−beta T cell activation
cell killing
T cell receptor signaling pathway
killing of cells of another organism
disruption of cell in another organism
B cell receptor signaling pathway
immunoglobulin mediated immune response
antigen receptor−mediated signaling pathway
B cell mediated immunity
antibacterial humoral response
antigen processing and presentation of exogenous peptide antigen via MHC class II
antigen processing and presentation of peptide antigen via MHC class II
antigen processing and presentation of peptide or polysaccharide antigen via MHC class II
antigen processing and presentation of exogenous peptide antigen
MHC class II protein complex assembly
positive regulation of phosphatidylinositol 3−kinase/protein kinase B signal transduction
regulation of phosphatidylinositol 3−kinase/protein kinase B signal transduction
phosphatidylinositol 3−kinase/protein kinase B signal transduction
regulation of angiogenesis
regulation of vasculature development
glomerular mesangium development
connective tissue development
muscle contraction
glomerulus vasculature development
renal system vasculature development
myeloid leukocyte activation
myeloid leukocyte mediated immunity
icosanoid metabolic process
prostaglandin metabolic process
prostanoid metabolic process
sample
Epithelium
Lymphoid
B_cells
Myeloid
Endothelium
Fibroblast
Mast_cells
Z−score
2
1
0
−1
−2
BC
Lymphoid
Fibroblast
Endothelium
Epithelium
Myeloid
B_cells
C1
C2
C3
C4
C5
C6
CD69
CD7
GZMK
COL3A1
COL6A3
PDGFRB
TPM2
TAGLN
CALD1
FN1
RAMP3
RAMP2
VWF
EGFL7
ESM1
ENG
S100A6
KRT7
KRT17
S100A2
FGF3
SNCG
CCND1
HLA−DRB1
HLA−DQB1
S100A8
APOC1
C15orf48
CD14
AIF1
FKBP11
IGHG1
CD79A
IGHA1
IGKV3−20
DERL3
IGLL5
IGHG3
MZB1
BANK1
positive thymic T cell selection
thymic T cell selection
alpha−beta T cell activation
cell killing
positive T cell selection
collagen fibril organization
connective tissue development
cellular response to amino acid stimulus
cartilage development
cellular response to acid chemical
positive regulation of angiogenesis
regulation of angiogenesis
positive regulation of vasculature development
regulation of vasculature development
vasculogenesis
intermediate filament organization
intermediate filament cytoskeleton organization
intermediate filament−based process
cell differentiation involved in embryonic placenta development
xenobiotic catabolic process
antigen processing and presentation of exogenous peptide antigen via MHC class II
antigen processing and presentation of peptide antigen via MHC class II
antigen processing and presentation of peptide or polysaccharide antigen via MHC class II
antigen processing and presentation of exogenous peptide antigen
antigen processing and presentation of exogenous antigen
B cell receptor signaling pathway
antigen receptor−mediated signaling pathway
antibacterial humoral response
immune response−activating cell surface receptor signaling pathway
immunoglobulin mediated immune response
sample
Lymphoid
Fibroblast
Endothelium
Epithelium
Myeloid
B_cells
Z−score
2
1
0
−1
−2
PCa
Myeloid
Epithelium
Fibroblast
Lymphoid
Endothelium
Mast_cells
B_cells
C1
C2
C3
C4
C5
C6
C7
Gene Size: 18
HLA−DRB5
HLA−DRB1
LYZ
CTSS
STEAP2
MSMB
GOLM1
TACSTD2
DSP
IGFBP5
ACTA2
MYL9
MYLK
RGS5
C11orf96
CCL2
IL32
CD7
CCL4
CXCR4
BTG1
CD69
EMCN
AQP1
EGFL7
RAMP3
ENG
ACSL4
IL1RL1
CPA3
LMNA
CD83
IRF8
LTB
IGKC
VPREB3
TNFRSF13C
CD79B
CCR7
CD37
antigen processing and presentation of exogenous peptide antigen via MHC class II
antigen processing and presentation of peptide antigen via MHC class II
antigen processing and presentation of peptide or polysaccharide antigen via MHC class II
antigen processing and presentation of exogenous peptide antigen
antigen processing and presentation of exogenous antigen
intermediate filament organization
intermediate filament cytoskeleton organization
intermediate filament−based process
feeding behavior
antimicrobial peptide production
muscle system process
muscle contraction
smooth muscle contraction
regulation of insulin−like growth factor receptor signaling pathway
negative regulation of cell motility
alpha−beta T cell activation
positive regulation of lymphocyte chemotaxis
regulation of lymphocyte chemotaxis
eosinophil chemotaxis
eosinophil migration
positive regulation of angiogenesis
positive regulation of vasculature development
vascular associated smooth muscle cell development
vasculogenesis
receptor internalization
myeloid cell activation involved in immune response
myeloid leukocyte activation
icosanoid metabolic process
prostaglandin metabolic process
prostanoid metabolic process
B cell receptor signaling pathway
antigen receptor−mediated signaling pathway
immune response−activating cell surface receptor signaling pathway
immune response−regulating cell surface receptor signaling pathway
lymphocyte differentiation
sample
Myeloid
Epithelium
Fibroblast
Lymphoid
Endothelium
Mast_cells
B_cells
Z−score
2
1
0
−1
−2

Figure S7. Heatmaps showing the top 20 marker genes of different cell types and their functional enrichment analyses.

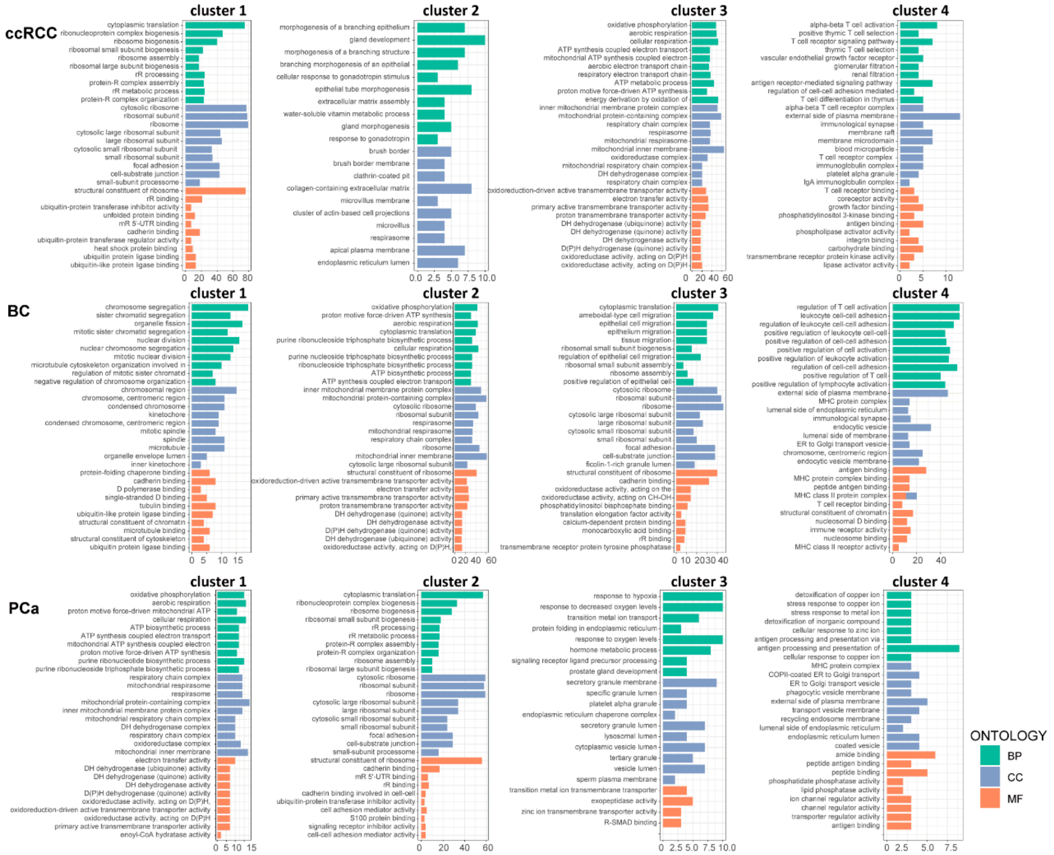

Figure S8. Functional enrichment during tumor cell differentiation.

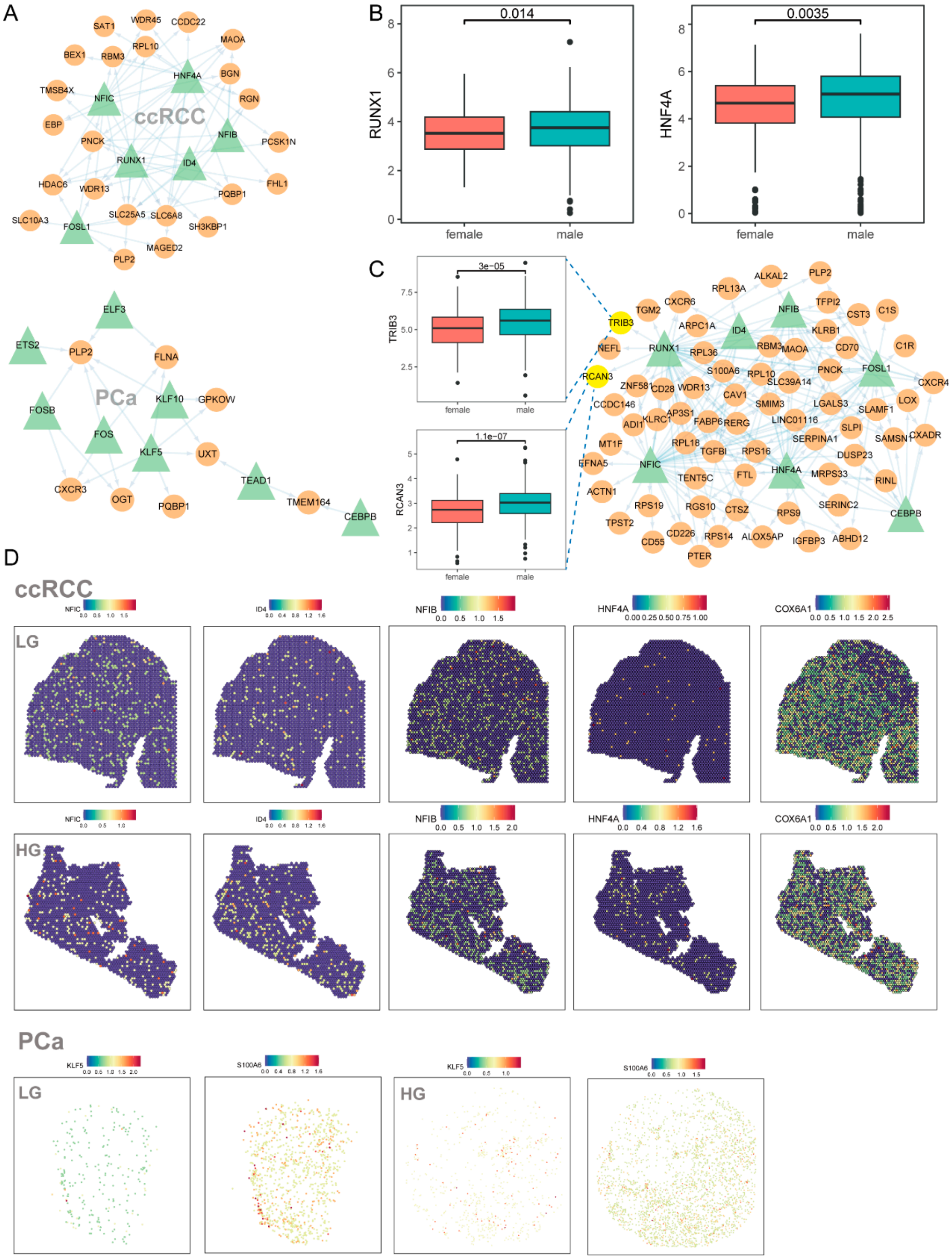

Figure S9. TFs regulatory network and gene expression in spatial locations.

(A) TFs–peak–gene networks of CNV genes on chromosomes X and Y. (B) Boxplots showing TFs RUNX1 and HNF4A exhibit higher expression levels in males. (C) TFs–peak–gene networks based on target genes showing higher expression in males. (D)

Spatial distribution of TFs and their target gene expression.

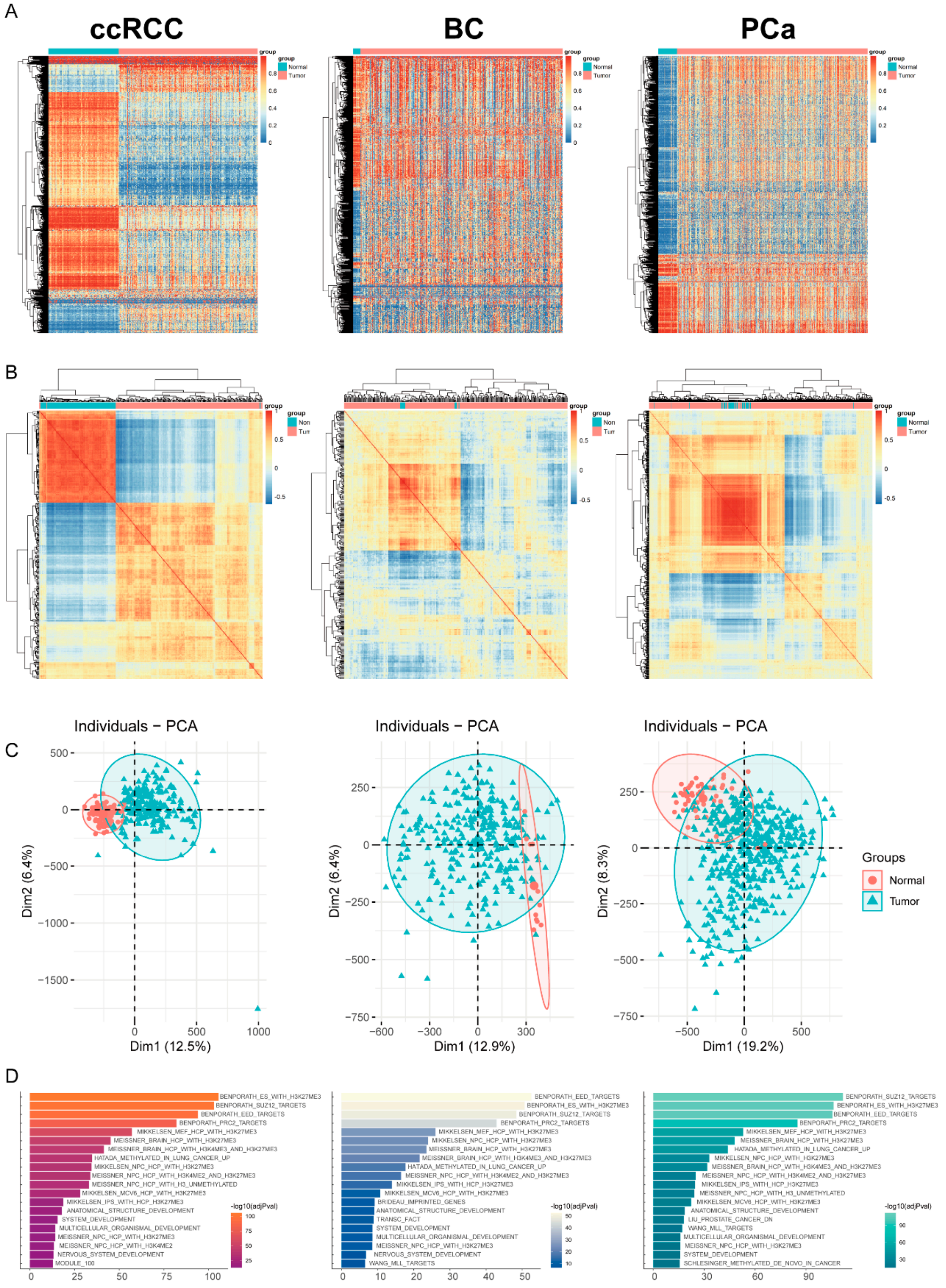

Figure S10. DNA methylation profiling in male urological cancers.

(A) Heatmaps showing differential methylation loci identified between normal and tumor tissues. (B) Hierarchical clustering of differentially methylated loci among

samples. (C) Scatter plot showing sample clustering based on PCA of differentially methylated loci. (D) Functional enrichment analysis of Differentially methylation.

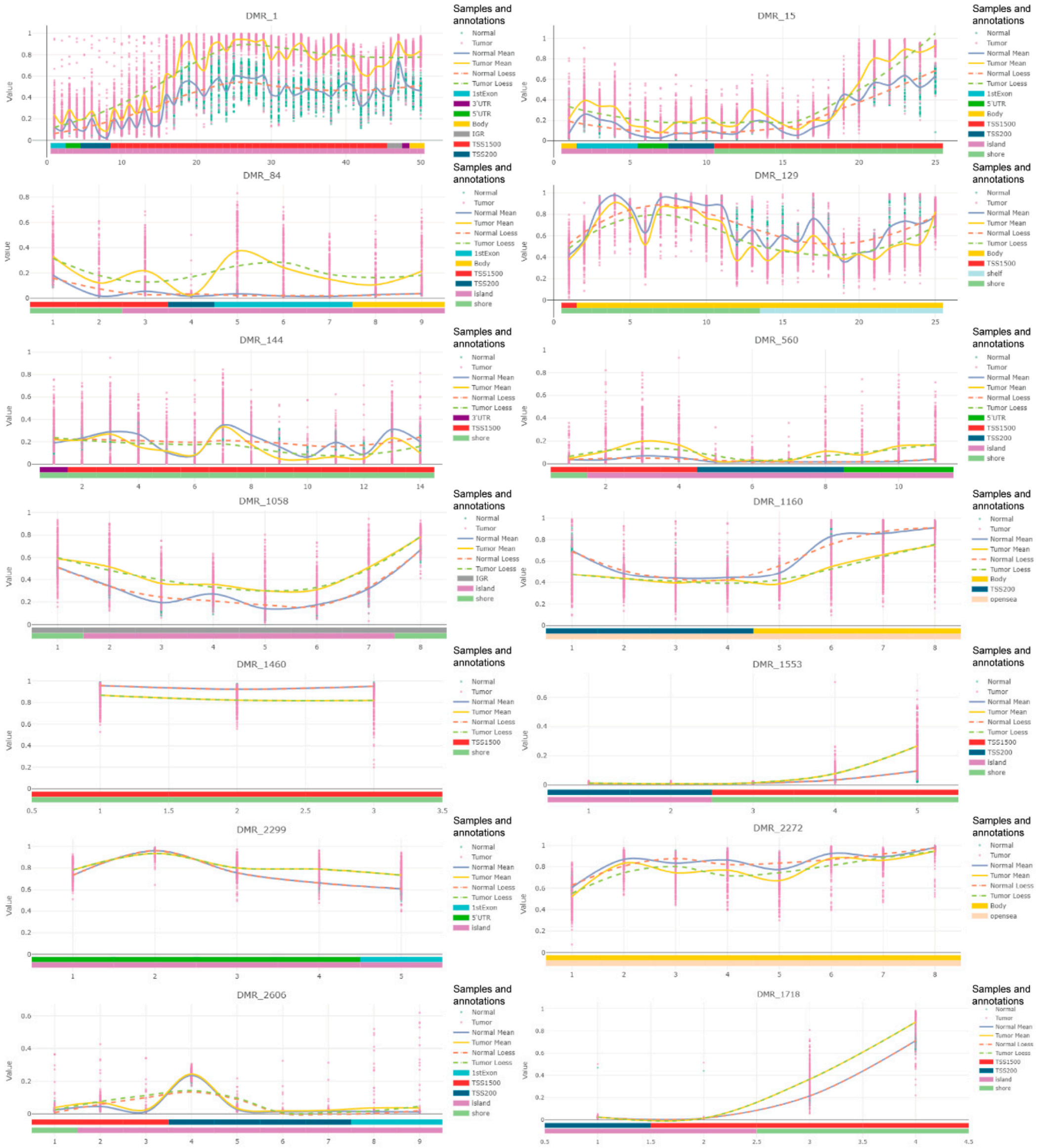

Figure S11. Line plot showing methylation regions overlapping with CNV regions in ccRCC.

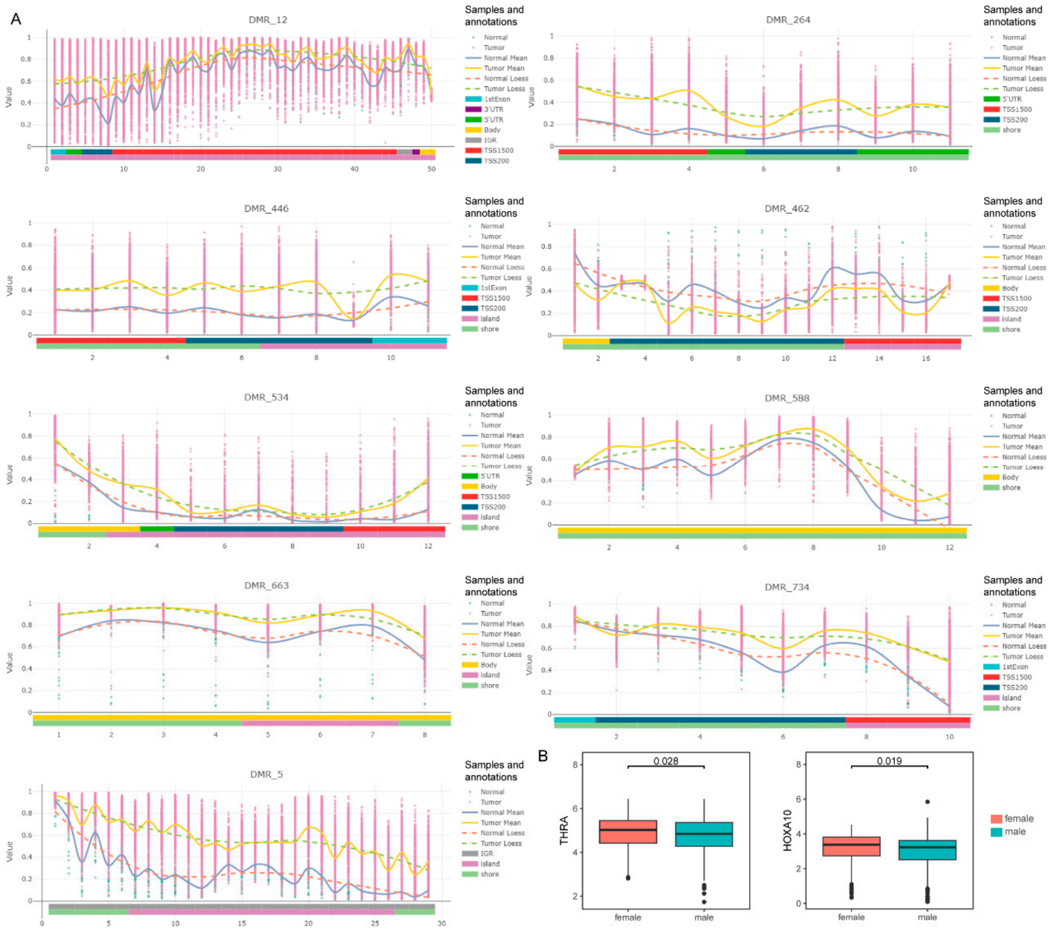

Figure S12. Line plot showing DMR regions overlapping with CNV regions in PCa.

(A) Line plot showing DMR regions overlapping with CNV regions in PCa. (B) Expression levels of THRA and HOXA10 in male and female patients with ccRCC in the TCGA dataset.

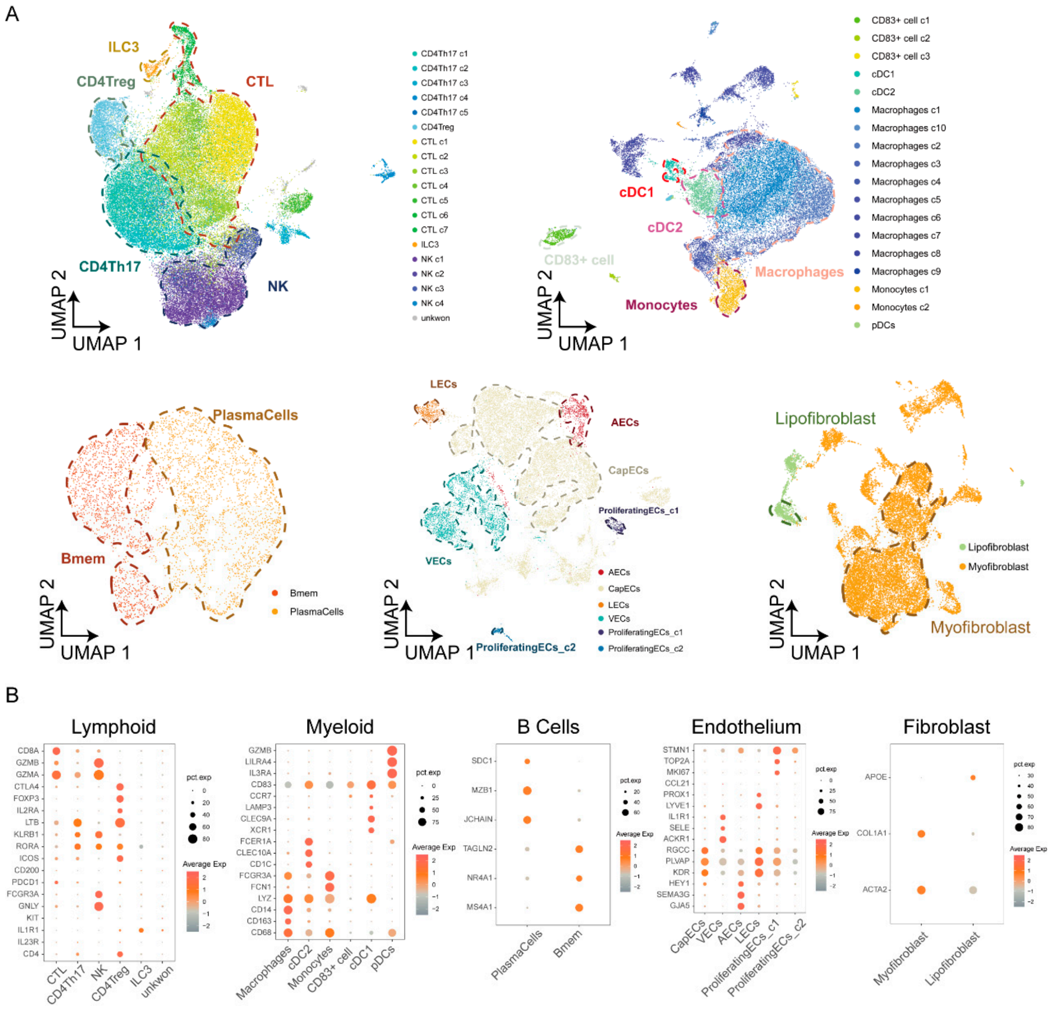

Figure S13. Clustering of cell subpopulations in male urological cancers.

(A) UMAP visualization of cellular subpopulation clusters within T cells, myeloid cells, B cells, endothelial cells, and fibroblasts. (B) Dot plots showing the expression of marker genes for T cells, myeloid cells, B cells, endothelial cells, and fibroblasts.

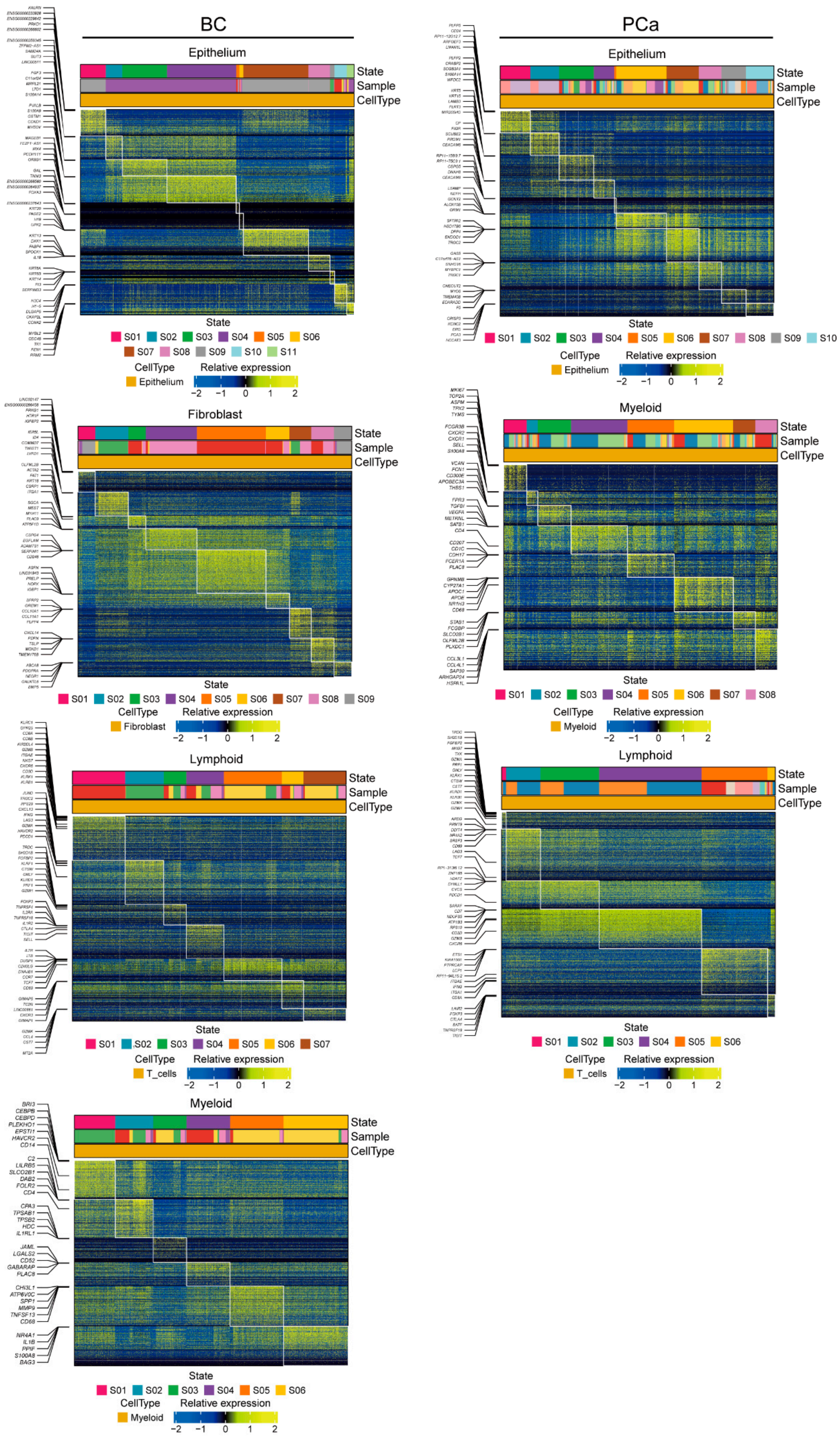
BC
PCa
Epithelium
State
Sample
CellType
S01 S02 S03 S04 S05 S06 S07 S08 S09 S10 S11
Relative expression
−2 −1 0 1 2
Epithelium
S01 S02 S03 S04 S05 S06 S07 S08 S09 S10
Fibroblast
S01 S02 S03 S04 S05 S06 S07 S08 S09
Myeloid
S01 S02 S03 S04 S05 S06 S07 S08
Lymphoid
S01 S02 S03 S04 S05 S06 S07
T_cells
S01 S02 S03 S04 S05 S06
Myeloid
BRI3
CEBPB
CEBPD
PLEKHO1
EPSTI1
HAVCR2
CD14
C2
LILRB5
SLCO2B1
DAB2
FOLR2
CD4
CPA3
TPSAB1
TPSB2
HDC
IL1RL1
JAML
LGALS2
CD52
GABARAP
PLAC8
CHI3L1
ATP6V0C
SPP1
MMP9
TNFSF13
CD68
NR4A1
IL1B
PPIF
S100A8
BAG3

Figure S14. Heatmaps showing cell states of various cell types in BC and PCa.

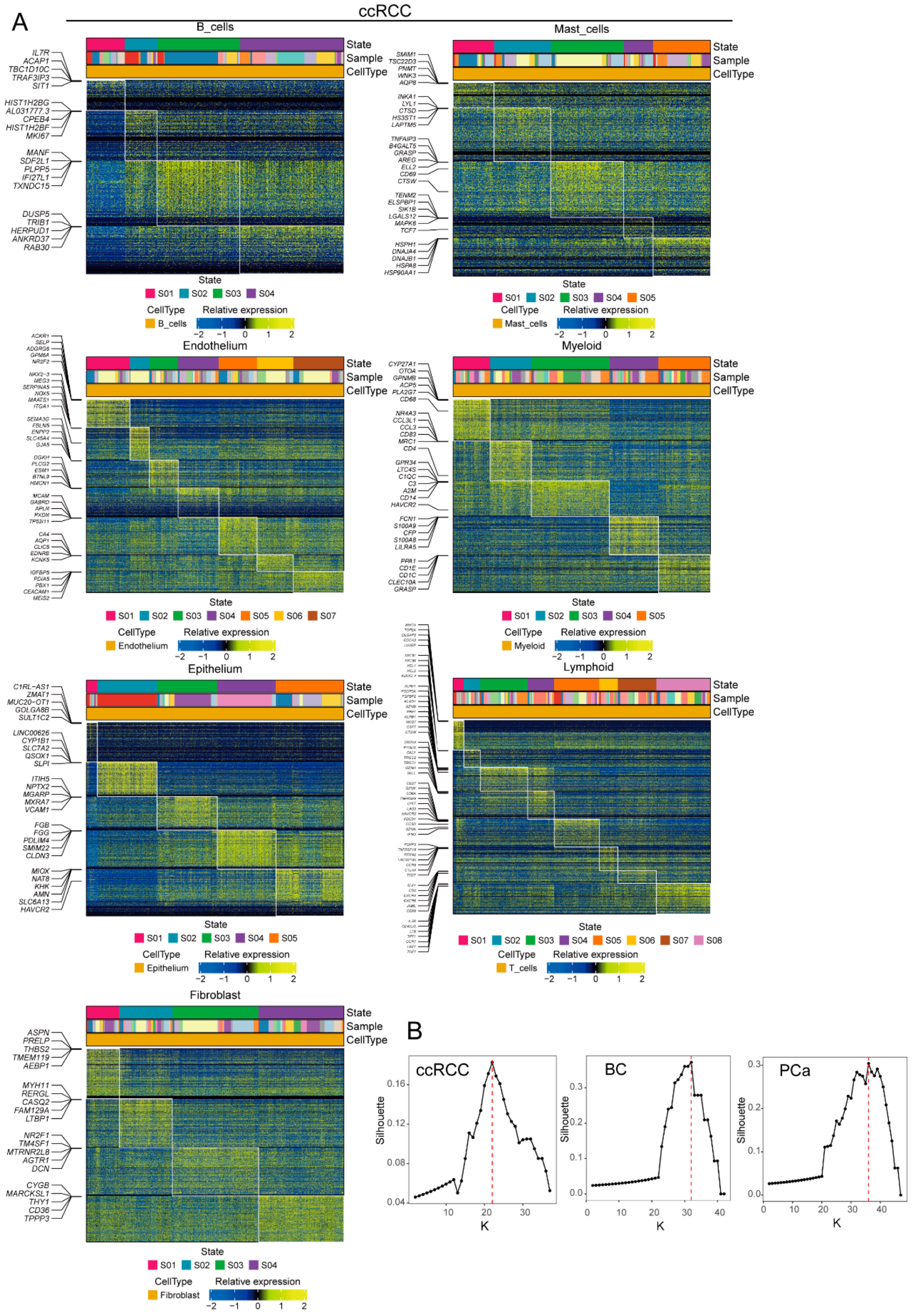

Figure S15. Identification of cellular states in male urological cancers.

(A) Heatmaps showing cell states of various cell types in ccRCC. (B) Plots displaying

the the number of initial clusters obtained by clustering the Jaccard index matrix, selected using the average silhouette

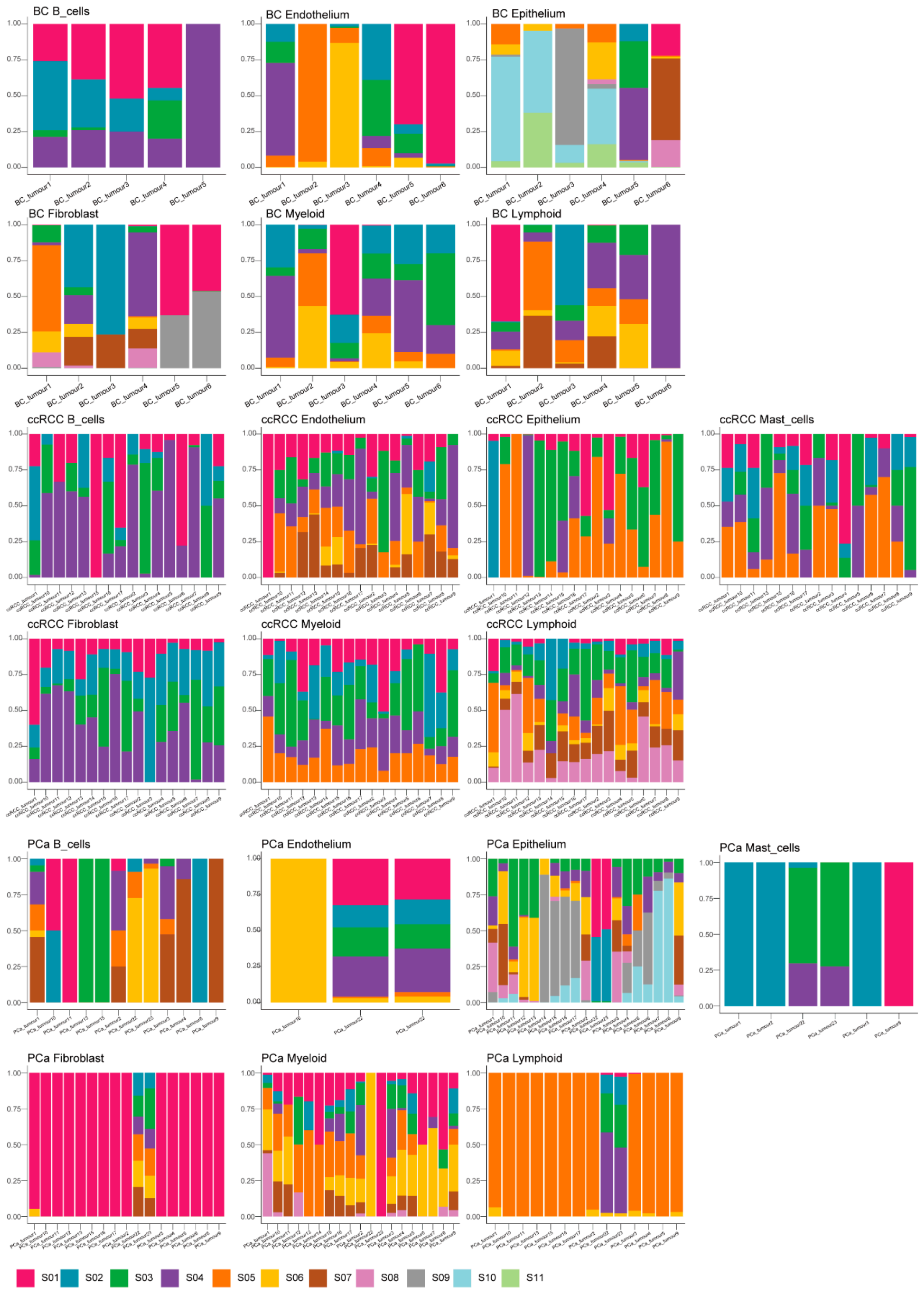

Figure S16. Proportion of cell states identified by EcoTyper in each sample.

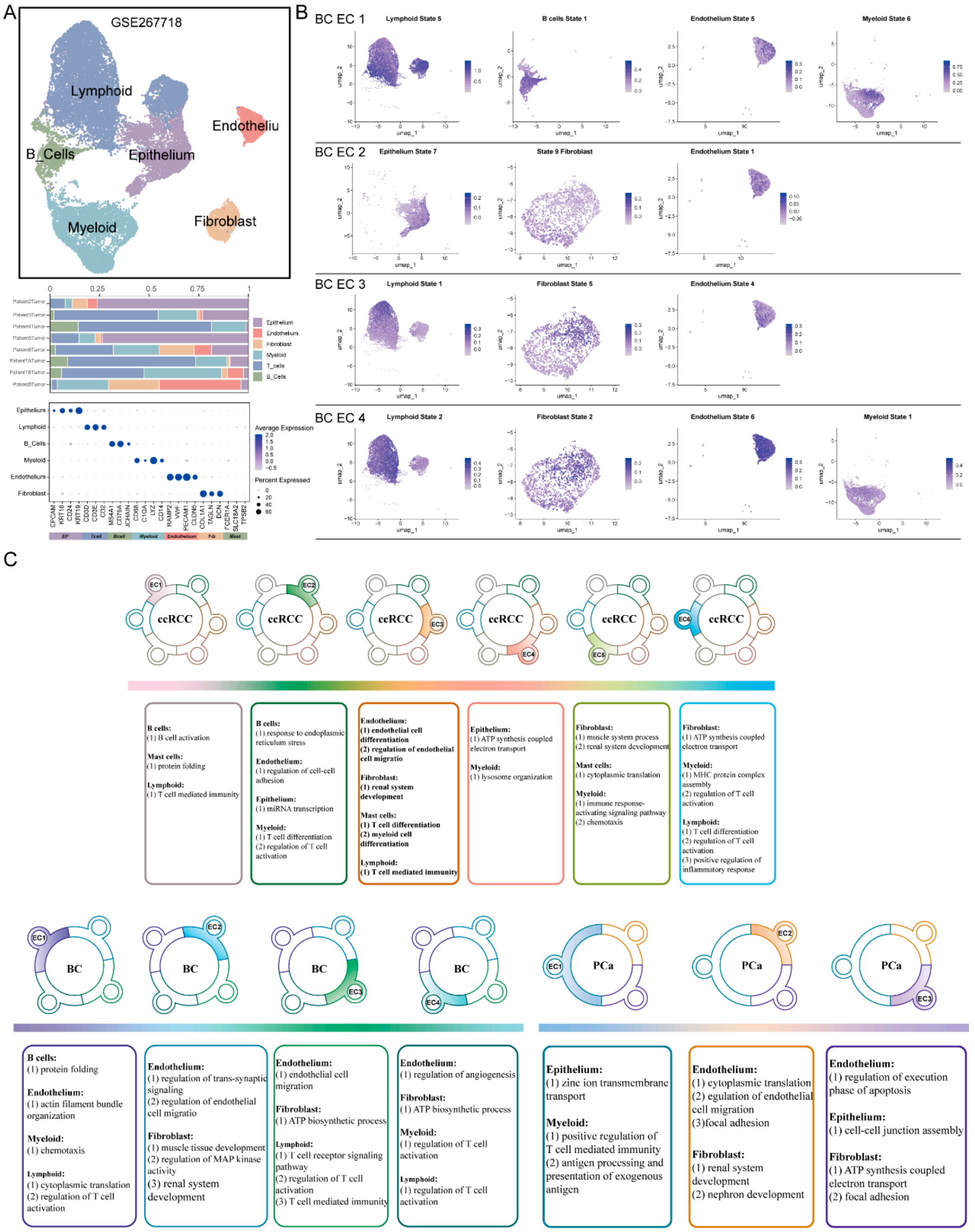

Figure S17. Functional enrichment analysis of cell states in male urological cancers.

(A) Dimensionality reduction and cell annotation of BC single-cell RNA-seq data from GSE267718. The bottom panels show cell type proportions per sample and marker gene expression for each cell type. (B) Scores of marker genes for cell states in BC ecotypes across different cell types in GSE267718. (C) Functional enrichment analysis of cell

states within each ecotype in three cancers.

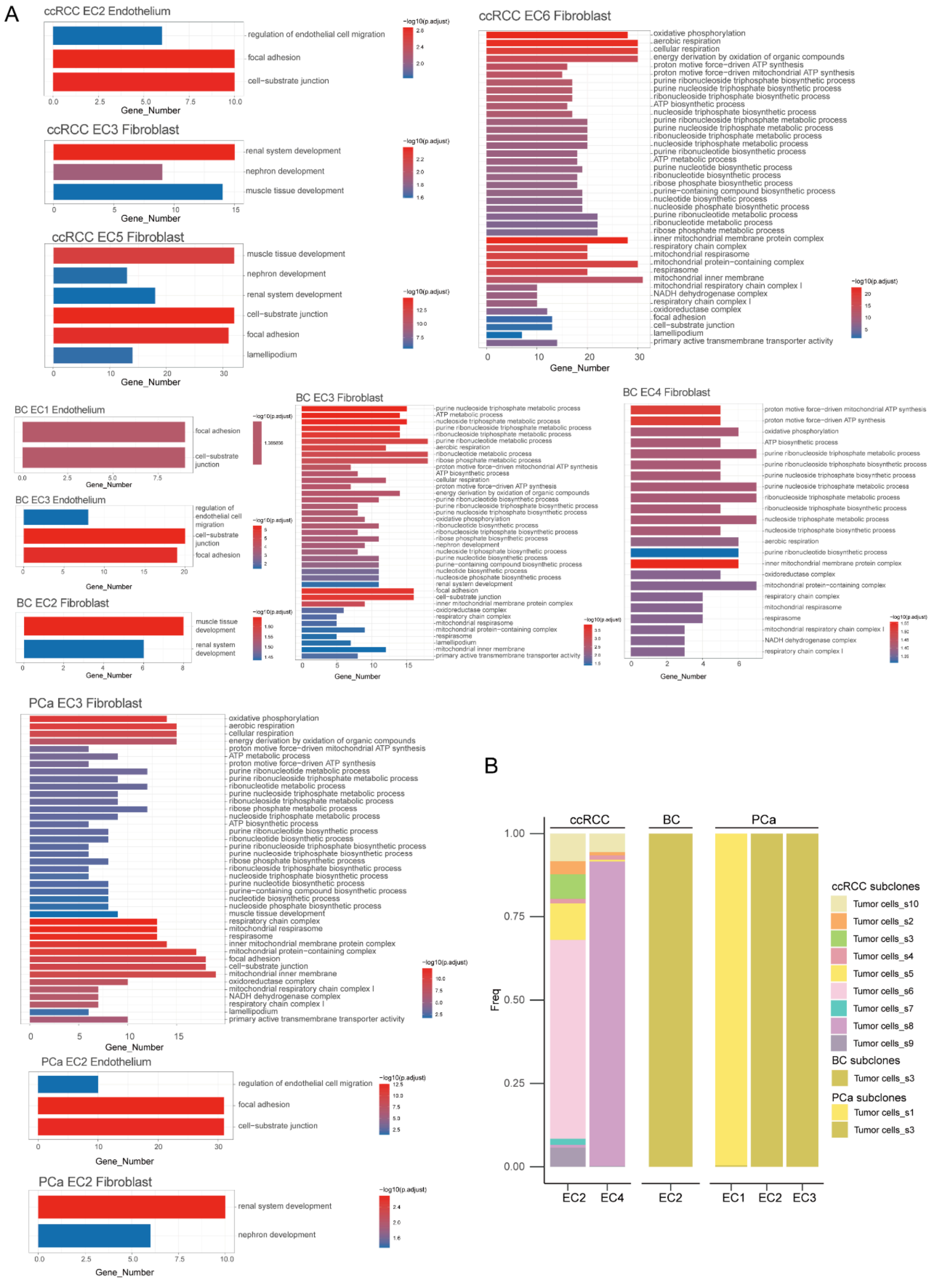

Figure S18. Functional enrichment analysis of cell states in stromal cells.

(A) Bar plots showing functional enrichment of endothelial cells and fibroblasts within each ecotype in three cancers. (B) The abundance of tumor cell subclones within tumor

cells across different ecotypes.

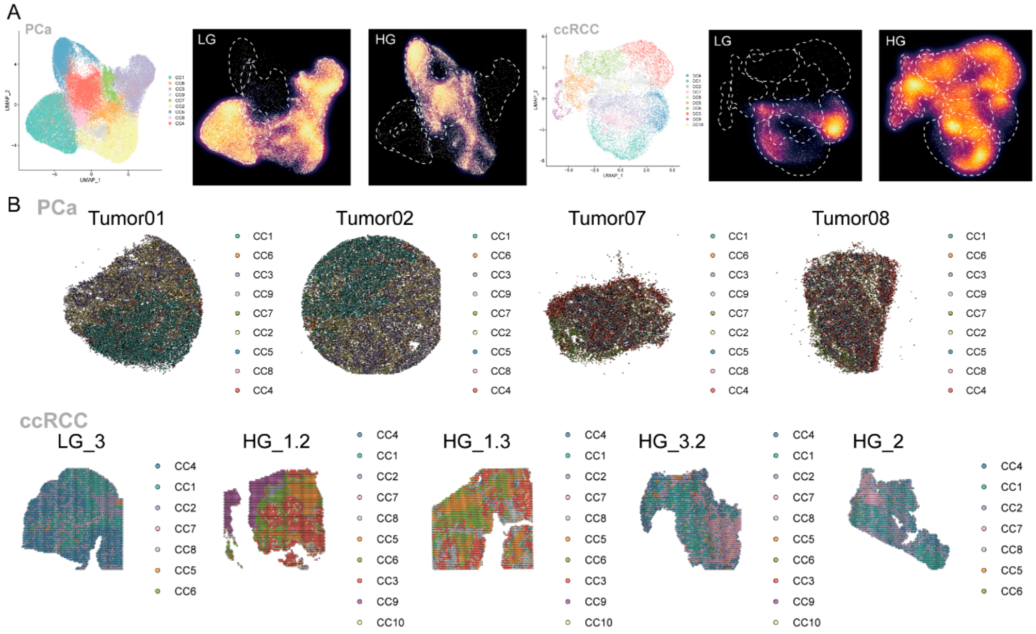

Figure S19. Integration of spatial transcriptomics data.

(A) Left: UMAP plot showing cell clustering after integration of spatial transcriptomics data from PCa samples. Spot density plots across gleason groups. Right: UMAP plot showing cell clustering after integration of spatial transcriptomics data from PCa samples. Spot density plots across different groups. (B) Spatial localization of compositional clusters (CCs) in ccRCC and PCa spatial transcriptomics.

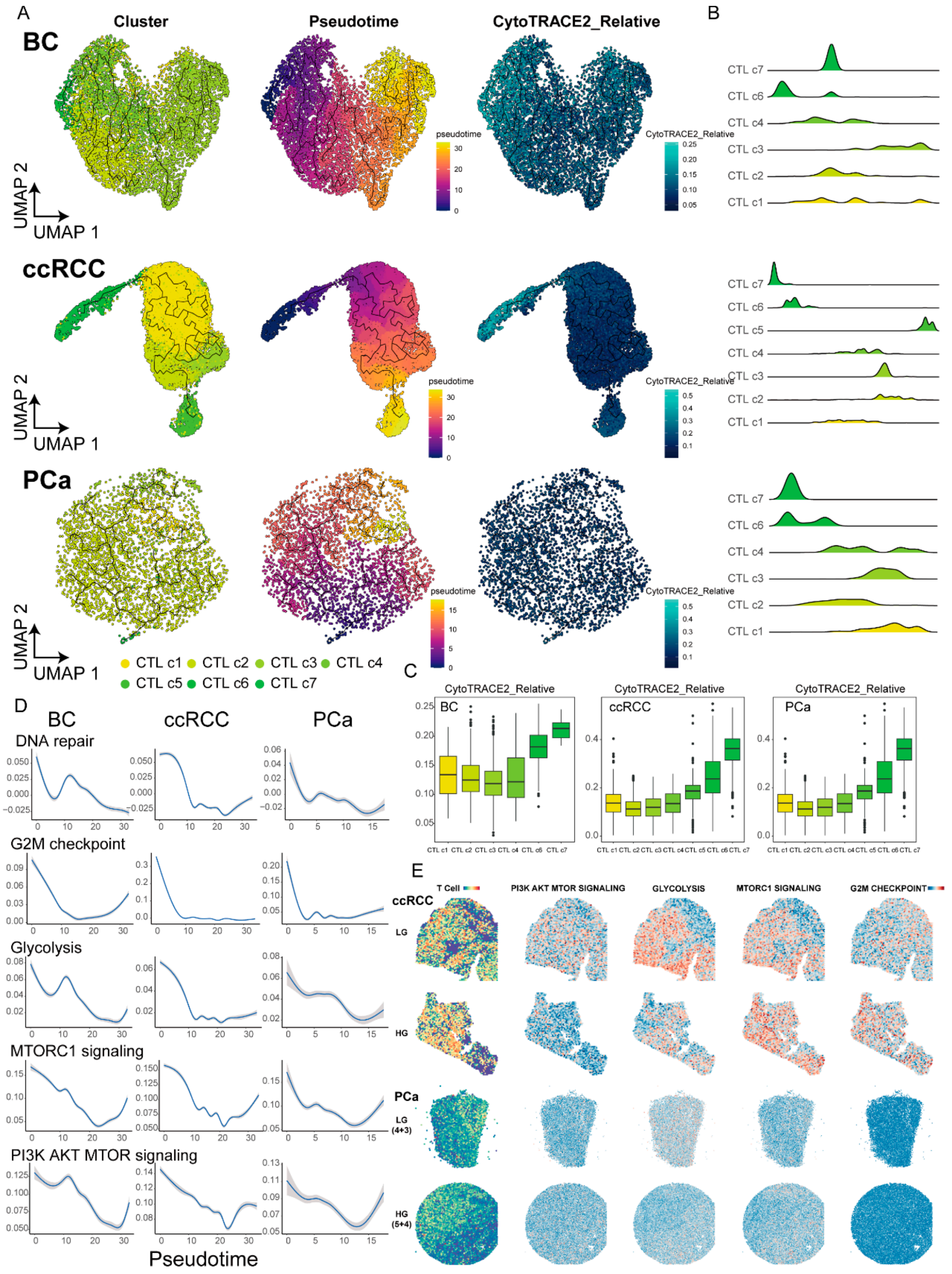

Figure S20. Pseudotime trajectory analysis of cytotoxic T lymphocyte (CTL).

(A) CTL differentiation trajectory in male urological cancers. (B) Ridge plots illustrating the distribution patterns of various cellular subpopulations throughout the differentiation process. (C) Boxplot showing stemness scores of cell subpopulations.

(D) Smoothed curves of cellular functional feature scores along pseudotime. (E) Scoring of gene sets in spatial transcriptomics spots and visualization of their spatial distribution.

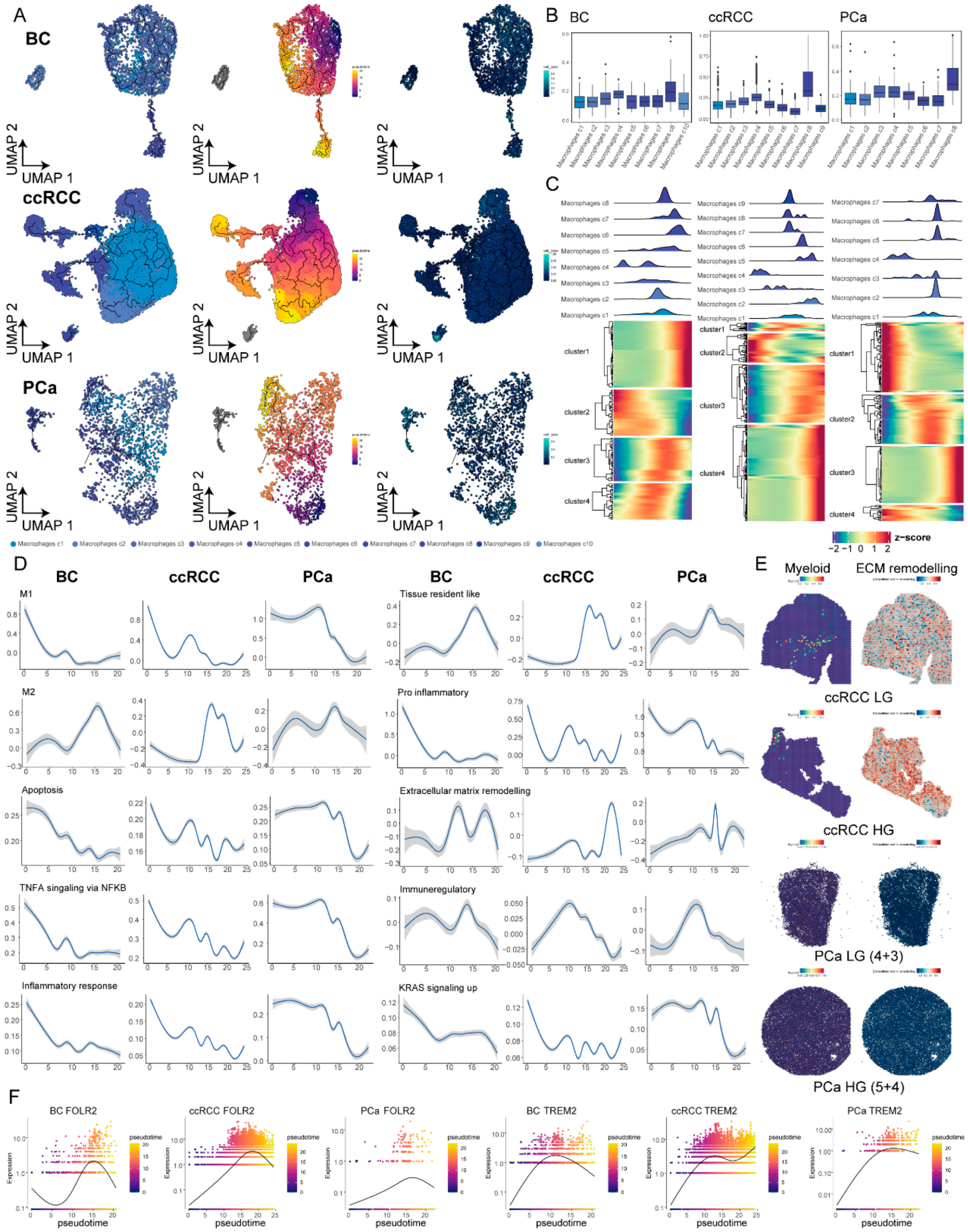

Figure S21. Pseudotime trajectory analysis of macrophages.

(A) Macrophages differentiation trajectory in male urological cancers. (B) Boxplot

showing stemness scores of cell subpopulations. (C) Ridge plots illustrating the distribution patterns of various cellular subpopulations throughout the differentiation process. Heatmaps illustrating gene clustering according to expression dynamics throughout cell differentiation. (D) Smoothed curves of cellular functional feature scores along pseudotime. (E) Scoring of gene sets in spatial transcriptomics spots and visualization of their spatial distribution. (F) Expression of macrophage markers FOLR2 and TREM2 along pseudotime.

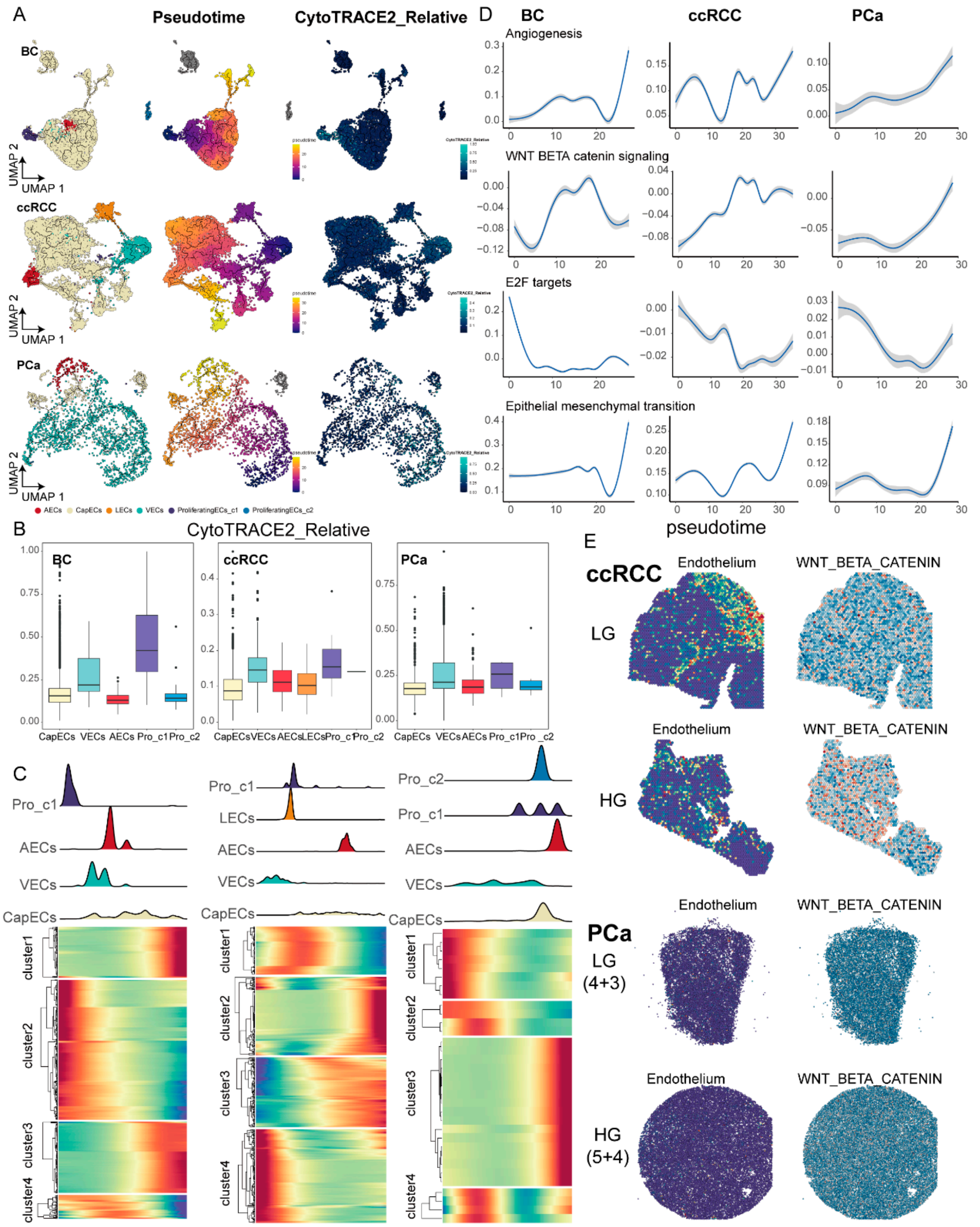

Figure S22. Pseudotime trajectory analysis of endothelial cells.

(A) Endothelial cell differentiation trajectory in male urological cancers. (B) Boxplot showing stemness scores of cell subpopulations. (C) Ridge plots illustrating the distribution patterns of various cellular subpopulations throughout the differentiation process. Heatmaps illustrating gene clustering according to expression dynamics

throughout cell differentiation. (D) Smoothed curves of cellular functional feature scores along pseudotime. (E) Scoring of gene sets in spatial transcriptomics spots and visualization of their spatial distribution.

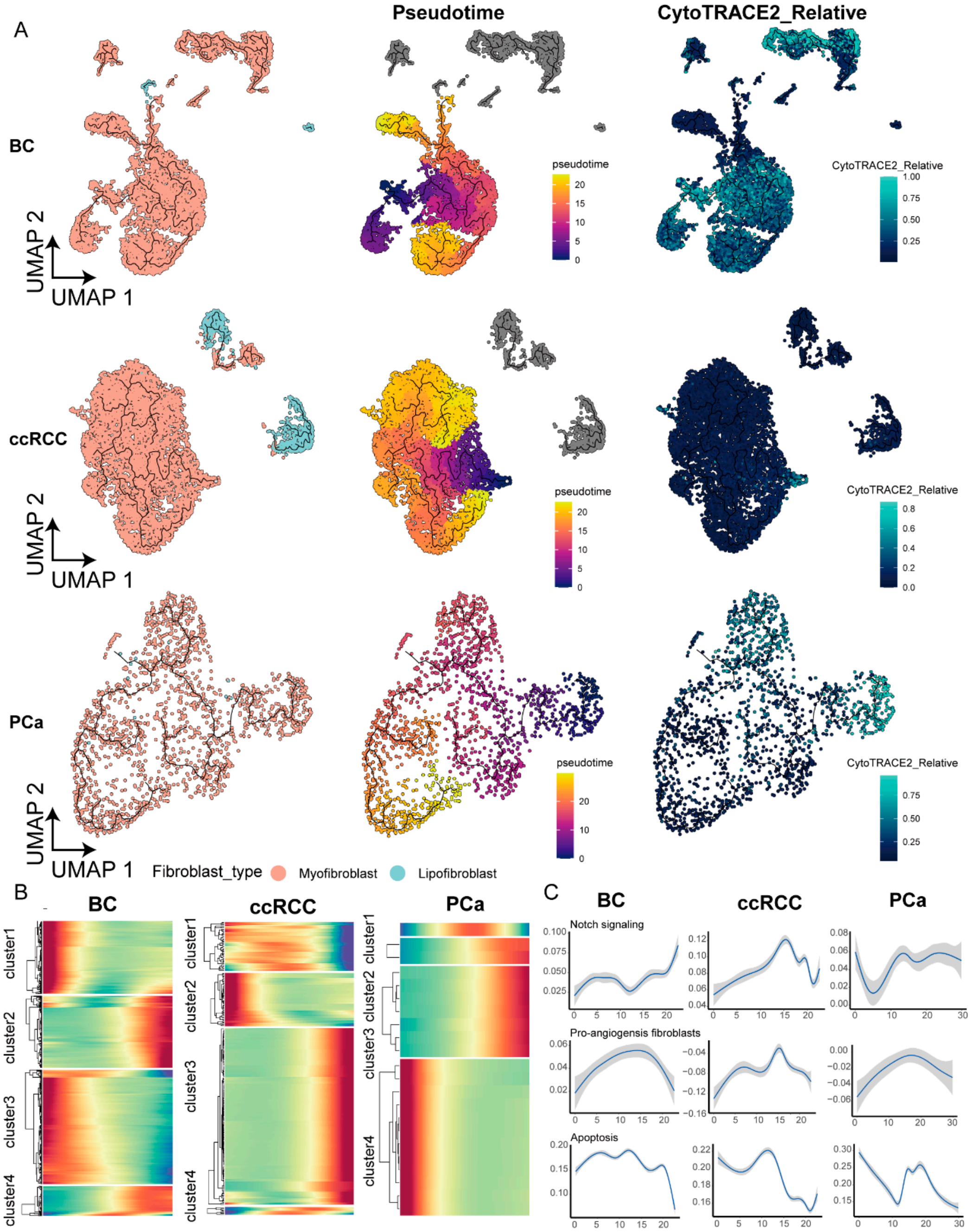

Figure S23. Pseudotime trajectory analysis of fibroblast.

(A) Fibroblast differentiation trajectory in male urological cancers. (B) Heatmaps

illustrating gene clustering according to expression dynamics throughout cell differentiation. (C) Smoothed curves of cellular functional feature scores along pseudotime.

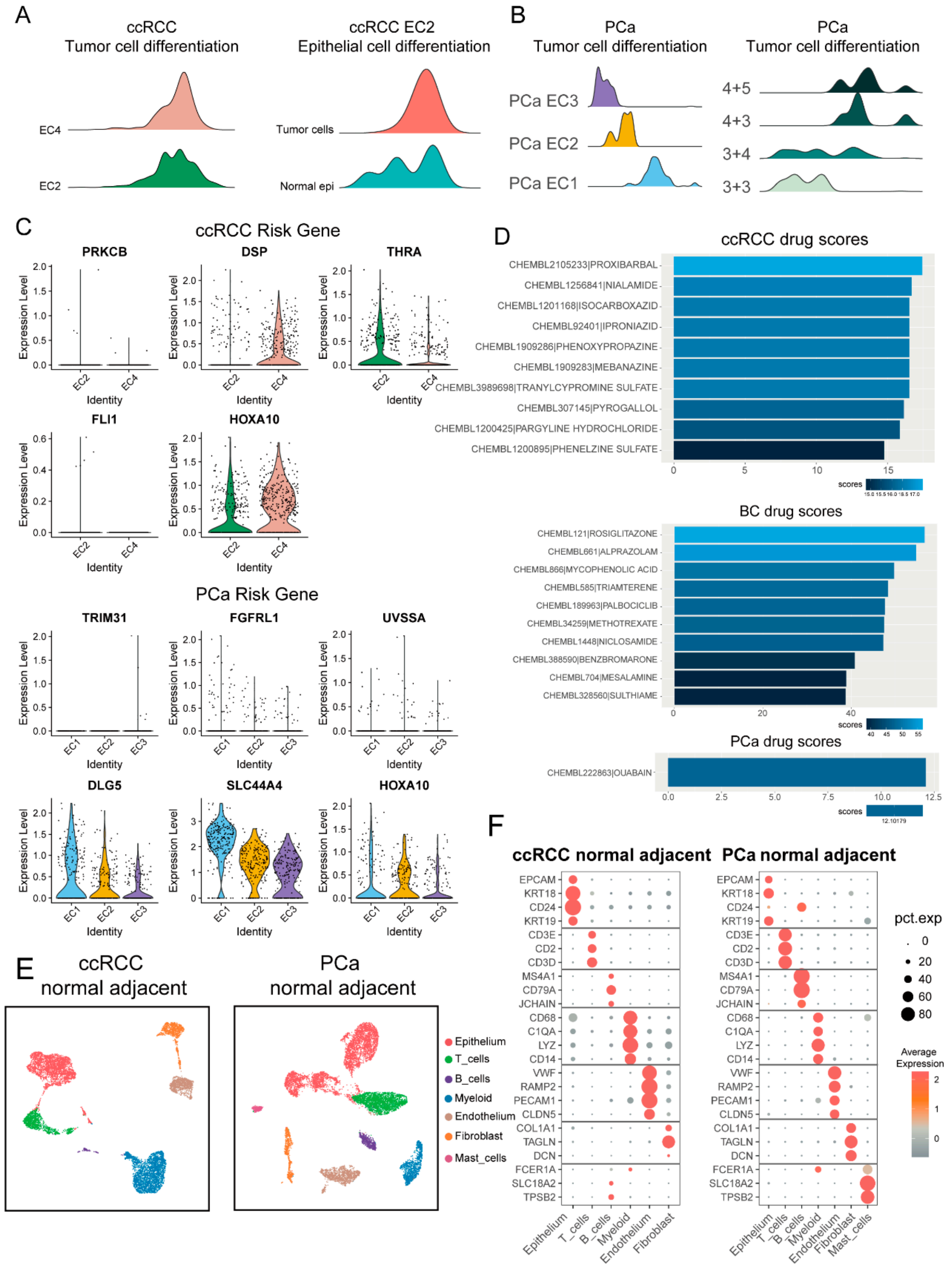

Figure S24. Differentiation of tumor cells in ecotypes and scRNA-seq data of

adjacent tissues.

(A) The ridge map shows the distribution of ecotypes and cell types during the differentiation process of ccRCC tumor cells. (B) The ridge map shows the distribution of ecotypes and cell types during the differentiation process of PCa tumor cells. (C) The violin plot displays the expression of risk genes in tumor cells of different ecotypes. (D) Bar plot showing drug scores targeting CNV genes on sex chromosomes. (E) UMAP visualization of cellular clusters derived from scRNA-seq data of adjacent normal tissues in ccRCC and PCa. (F) Dot plot showing expression of marker genes for cell types in adjacent normal tissues.
